# Comparative genomic analysis and phylogenetic position of *Theileria equi*

**DOI:** 10.1186/1471-2164-13-603

**Published:** 2012-11-09

**Authors:** Lowell S Kappmeyer, Mathangi Thiagarajan, David R Herndon, Joshua D Ramsay, Elisabet Caler, Appolinaire Djikeng, Joseph J Gillespie, Audrey OT Lau, Eric H Roalson, Joana C Silva, Marta G Silva, Carlos E Suarez, Massaro W Ueti, Vishvanath M Nene, Robert H Mealey, Donald P Knowles, Kelly A Brayton

**Affiliations:** 1 Animal Disease Research Unit, Agricultural Research Service, USDA, Pullman, WA, 99164-7030, USA; 2 J. Craig Venter Institute, Rockville, MD, 20850, USA; 3 Department of Veterinary Microbiology & Pathology, Washington State University, Pullman, WA, 99164-7040, USA; 4 International Livestock Research Institute, P.O. Box 30709, Nairobi, 00100, Kenya; 5 Virginia Bioinformatics Institute at Virginia Tech, Blacksburg, VA, 24061, USA; 6 Institute for Genome Sciences and Department of Microbiology and Immunology, University of Maryland School of Medicine, Baltimore, MD, 21201, USA; 7 Paul G. Allen School for Global Animal Health, Washington State University, Pullman, WA, 99164-7040, USA; 8 School of Biological Sciences, Washington State University, Pullman, WA, 99164-4236, USA; 9 Current address: Frederick National Lab for Cancer Research, Rockville, MD, 20852, USA

**Keywords:** Apicomplexa, Parasite, Vaccine, Horse, Vector-borne disease

## Abstract

**Background:**

Transmission of arthropod-borne apicomplexan parasites that cause disease and result in death or persistent infection represents a major challenge to global human and animal health. First described in 1901 as *Piroplasma equi*, this re-emergent apicomplexan parasite was renamed *Babesia equi* and subsequently *Theileria equi*, reflecting an uncertain taxonomy. Understanding mechanisms by which apicomplexan parasites evade immune or chemotherapeutic elimination is required for development of effective vaccines or chemotherapeutics. The continued risk of transmission of *T*. *equi* from clinically silent, persistently infected equids impedes the goal of returning the U. S. to non-endemic status. Therefore comparative genomic analysis of *T*. *equi* was undertaken to: 1) identify genes contributing to immune evasion and persistence in equid hosts, 2) identify genes involved in PBMC infection biology and 3) define the phylogenetic position of *T*. *equi* relative to sequenced apicomplexan parasites.

**Results:**

The known immunodominant proteins, EMA1, 2 and 3 were discovered to belong to a ten member gene family with a mean amino acid identity, in pairwise comparisons, of 39%. Importantly, the amino acid diversity of EMAs is distributed throughout the length of the proteins. Eight of the EMA genes were simultaneously transcribed. As the agents that cause bovine theileriosis infect and transform host cell PBMCs, we confirmed that *T*. *equi* infects equine PBMCs, however, there is no evidence of host cell transformation. Indeed, a number of genes identified as potential manipulators of the host cell phenotype are absent from the *T*. *equi* genome. Comparative genomic analysis of *T*. *equi* revealed the phylogenetic positioning relative to seven apicomplexan parasites using deduced amino acid sequences from 150 genes placed it as a sister taxon to *Theileria spp*.

**Conclusions:**

The EMA family does not fit the paradigm for classical antigenic variation, and we propose a novel model describing the role of the EMA family in persistence. *T*. *equi* has lost the putative genes for host cell transformation, or the genes were acquired by *T*. *parva* and *T*. *annulata* after divergence from *T*. *equi*. Our analysis identified 50 genes that will be useful for definitive phylogenetic classification of *T*. *equi* and closely related organisms.

## Background

Equine piroplasmosis of horses, mules, donkeys and zebras is caused by the tick-borne apicomplexan protozoan parasites *Babesia caballi* and *Theileria equi*, transmitted by ixodid ticks such as *Dermacentor nitens* (*B*. *caballi*) and *Rhipicephalus microplus* (*T*. *equi*) [1,2]. Although endemic in most countries [3], the U. S., until recently, has been considered free of infection. Equine infections in Florida with *B*. *caballi* and *T*. *equi* were diagnosed between 1961 and 1969 leading to an eradication campaign which lasted twenty-five years and cost twelve million dollars [4]. The re-emergence of *T*. *equi* in Florida [4] and Texas [5] raised concern of its further spread within the U. S., and indeed, infected horses have been identified in 12 states [6,7]. The cause of the 2008 Florida outbreak was due to iatrogenic transmission, but two tick species, *Amblyomma cajennense* and *D*. *variabilis*, were identified as novel vectors in the 2009 Texas outbreak [5]. The re-emergence of this pathogen in the U.S. impacts global movement and health of horses and affects the multi-billion dollar equine industry.

Additional members of the phylum Apicomplexa, important to global human and animal health include the organisms in the genus *Plasmodium* as well as *T*. *parva* and *T*. *annulata*, and *Babesia bovis* causes of malaria, bovine theileriosis and babesiosis, respectively. The phylogenetic position of *T*. *equi* has been controversial, and the organism has been renamed several times [8]. Molecular phylogenetic analyses indicate an intermediate position for *T*. *equi* between *B*. *bovis* and *Theileria* spp. [9,10] and is supported by the genomic data presented here which provides the deepest phylogenetic analysis to date. Collective data supports the concept that a new genus placement sister to *Theileria* may be appropriate for *T*. *equi*.

Similar to bovine theileriosis caused by *T*. *annulata*, transmission of *T*. *equi* to equids eventually results in lysis of erythrocytes and prolonged anemia. Anemia associated with *T*. *parva* occurs later during infection and is comparatively and clinically mild [11]. Infection of B- and T-lymphocytes by *T*. *parva* and mononuclear phagocytes and B-lymphocytes by *T*. *annulata* lead to reversible cell transformation [12]. Infection of peripheral blood mononuclear cells (PBMCs) by *T*. *equi* has been reported [8,13]. However the role of PBMC infection in the pathogenesis of *T*. *equi*, unlike *T*. *parva* and *T*. *annulata* remains unresolved, and PBMC proliferation and/or transformation have not been associated with clinical equine piroplasmosis.

The primary clinical outcome of acute *T*. *equi* infection is anemia and the associated erythrolysis is independent of parasite-specific immune responses [14]. Resolution of acute disease is followed by apparent life-long parasite persistence within equids [15]. Persistence is characterized by the continuous presence of 10^3^ to 10^6^ infected peripheral erythrocytes per ml/blood resulting in efficient acquisition and transmission by ticks [16]. A hallmark of pathogens that establish persistent infection and avoid immune elimination is the presence of an immunodominant, variable multigene family responsible for immune evasion, such as VESA1 (Variant Erythrocyte Surface Antigen 1) in *B*. *bovis*[17], PfEMP1 (Erythrocyte Membrane Protein 1) in *P*. *falciparum*[18] and VSG in *T*. *brucei*[19]. An analogous family was not detected in *T*. *equi*. A candidate multigene gene family in *T*. *equi* encodes Equi Merozoite Antigens (EMAs), which are immunodominant for antibody, however, this family contains just 10 members, and lacks an apparent structural basis for variation. The reemergence and persistence of *T*. *equi* in the U. S. prompted this genomic research due to the lack of a vaccine to block infection or clinical disease and the need for additional chemotherapeutics aimed at eliminating persistent infection and tick-borne transmission risk.

## Results and discussion

### Chromosome arrangement and content

**Figure 1:**
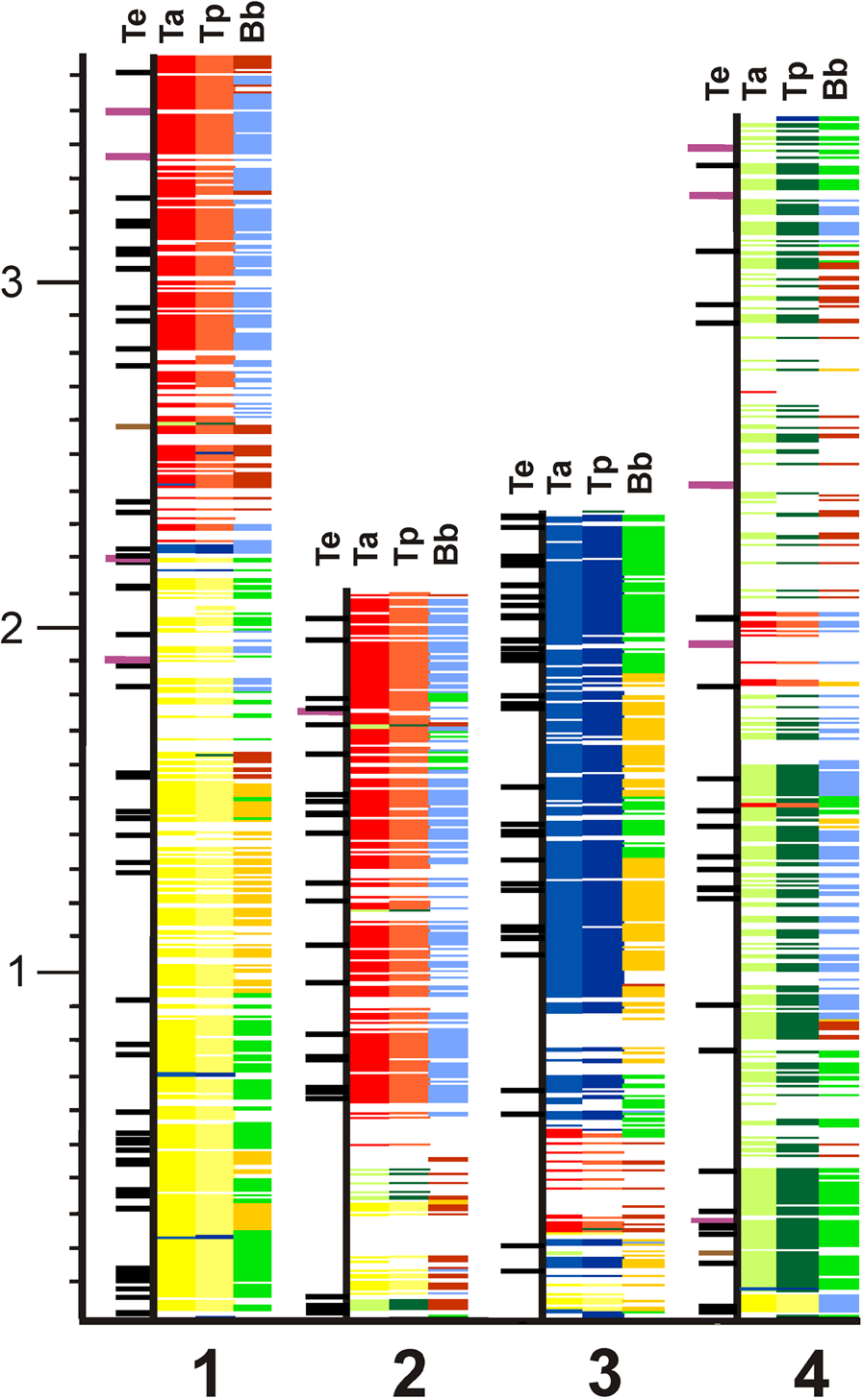
**Chromosomal map and depiction of synteny between piroplasms. ***T*. *equi* (Te), *T*. *annulata* (Ta), *T*. *parva* (Tp) and *B*. *bovis* (Bb) chromosomes are shown with the *T*. *equi* chromosome represented by the thin black line, and corresponding syntenic regions from other species’ chromosomes shown as color blocks. *S*hades of red represent chromosomes 1, shades of yellow represent chromosomes 2, shades of green represent chromosomes 3, and shades of blue represent chromosomes 4. To the left of the Te chromosome are indicated genes discussed in the manuscript: purple bars represent locations of *ema* family members, black bars represent the location of genes used in phylogenetic analysis and brown bars represent the two rRNA operons.

The genomic complement of *T*. *equi* contains six molecules, including four chromosomes (Figure 1) of ~11.6 Mbp, an apicoplast genome of 47.8 kbp and a mitochondrial genome of 9 kbp. Chromosomes 1 (3,677,484 bp) and 3 (2,338,319 bp) were completely assembled, while chromosome 2 (2,060,349 bp) contains one assembly gap. Chromosome 4 (3,480,987 bp) is fragmented into six pieces with four physical gaps and one assembly gap. The chromosome assemblies agree with the sizes of the chromosomes seen on pulsed field gel electrophoresis, suggesting that there are no large gaps in the genome sequence.

The larger genome size of *T*. *equi* as compared to other piroplasms (*Theileria* and *Babesia*) (Table 1) is also reflected in the number of predicted protein coding genes (5330), ~ 25% greater than found for *T*. *parva*, *T*. *annulata* and *B*. *bovis*. While *T*. *equi* contains homologs of genes found only in the two *Theileria* spp. (366), or *B*. *bovis* (137), it has far more unique genes (1985), which account for the increased size of the genome. The four species have 2,482 genes in common. *T*. *equi* has two rRNA operons, and 46 tRNA genes.

**Table 1:** Genome characteristics of Apicomplexans

		**Species**^**a**^			
**Features**	***Pf***	***Tp***	***Ta***	***Bb***	***Te***
Size (Mbp)	22.8	8.3	8.35	8.2	11.6
Number of chromosomes	14	4	4	4	4
Total G+C composition (%)	19.4	34.1	32.5	41.8	39.5
Size of apicoplast genome (kbp)	35	39.5	Nr^e^	33	47.8
Size of mitochondrial genome (kbp)^b^	6 L	6 L	6L	6 L	9 L
# of nuclear protein coding genes	5268	4035	3807	3670	5330
Average CDS length (bp)^c^	2283	1407	1600	1514	1472
Percent genes with introns	53.9	73.6	70.6	61.5	52.4
Mean length of intergenic region (bp)	1694	405	396	589	550
G+C composition of intergenic region	13.8	26.2	24.1	37	39.3
G+C composition of exons (%)	23.7	37.6	35.7	44	39.8
G+C composition of introns (%)	13.6	25.4	24.4	35.9	37.6
Percent coding	52.6	68.4	72.9	70.2	69.3
Gene density^d^	4338	2057	2195	2228	2185

^a^ Pf = *P*. *falciparum*, Tp = *T*. *parva*, Ta = *T*. *annulata*, Bb = *B*. *bovis*, Te = *T*. *equi.*

^b^ L indicates the presence of a linear genome.

^c^ not including introns.

^d^ genome size/number of protein coding genes.

^e^ not reported.

### The apicoplast

The apicoplast is a plastid-like organelle thought to be derived from a secondary endosymbiotic event with green algae [20,21]. Like plastids, many of the genes for metabolic processes in the apicoplast have migrated to the nuclear genome leaving a remnant genome [22]. In *T*. *equi*, the A+T rich (71%) 47.8 kb apicoplast genome is larger than those of other piroplasms, due primarily to expansion of the repertoires of three hypothetical genes (Additional file 1). There are 43 unidirectionally encoded CDSs in the *T*. *equi* molecule, which includes 11 ribosomal protein coding sequences. Additionally, each of the 20 tRNA and two rRNA genes are present.

*T*. *equi* contains 509 nuclear-encoded proteins potentially targeted to the apicoplast as predicted by PlasmoAP [23], ApicoAP [24] and/or by homology to genes for pathways predicted to occur in the apicoplast (Additional file 2). Similar to other apicomplexans, *T*. *equi* has a complete set of nuclear-encoded enzymes for isoprenoid precursor biosynthesis via the methylerythritol phosphate pathway and these activities are predicted to occur in the apicoplast.

### Mitochondrial genome

The mitochondrial (mt) genome is 9001 bp in length, longer than in other piroplasms and shows evidence of gene duplication and rearrangement. The *T*. *equi* mt sequence was recently reported and the authors suggest that a duplicated CDS (BEWA_044660 and BEWA_044650) is actually cytochrome c oxidase subunit III (*cox3*) [25]. Although there is no sequence similarity to known *cox3* sequences, *T*. *equi* could not conduct respiration without *cox3*, and these genes are the only candidate for this function. Our findings corroborate the linear structure of the mt genome and the long inverted terminal repeat structure [25].

### Metabolism

#### Energy Production

The predicted metabolic profile of *T*. *equi* is similar to other piroplasms: most elements of the core pathways for energy production are present including glycolysis, pentose phosphate pathway and the tricarboxylic acid (TCA) cycle. Glycolysis is fully intact with putative enzymes identified for each stage of the conversion of sugars to pyruvate for the production of energy, making carbon the primary energy source. However, since pyruvate dehydrogenase (EC 1.2.4.1) is missing from the *T*. *equi* genome (similar to *B*. *bovis* and other *Theileria* spp.), glycolysis does not seem to be coupled to the TCA cycle. Although the TCA cycle is intact, the lack of pyruvate dehydrogenase suggests that the primary function may be to generate precursors rather than produce energy. For example, succinyl CoA is a critical biosynthetic precursor for the synthesis of protoheme, used in cytochromes and many enzymes. Electron transport pathways in *T*. *equi* resemble those reported for *P*. *falciparum*[26]. Lack of the full complement of NADH dehydrogenase subunits and ATP synthase peptides makes it questionable as to whether the electron transport pathway is efficient for the generation of ATP from the products of glycolysis.

#### Small molecule synthesis

Metabolic pathway similarities with other sequenced hemoparasites include the ability to synthesize pyrimidines, limited amino acid biosynthesis, and the lack of a urea cycle. *De novo* purine biosynthesis is absent, however, unlike other piroplasms, hypoxanthine/guanine phosphoribosyl transferase (BEWA_017710) and adenine phosphoribosyltransferase (BEWA_017730) are present, indicating that purine salvage can occur, similar to *P*. *falciparum*. Fatty acid synthesis, heme biosynthesis and the shikimic acid pathway do not occur in the piroplasms, although these pathways are present in *P*. *falciparum*[26-29].

*T*. *equi* encodes dihydrofolate synthase (EC 6.3.2.17, BEWA_029790) and a bifunctional dihydrofolate reductase-thymidylate synthase (EC 1.5.1.3, DHFR-TS, BEWA_008170) and is predicted to carry out a limited folate biosynthetic pathway similar to *Theileria* spp [27,28]. Notably, a large number of folate-biopterin transporters were predicted, suggesting that *T*. *equi* imports folate which is then modified into other compounds, chiefly nucleotide precursors. Folate biosynthesis predicts sensitivity to the drug pyrimethamine cycloguaryl, which has been observed in previous studies [30], even though *T*. *equi* DHFR-TS encodes the S125F mutation that reportedly confers pyrimethamine resistance in *B*. *bovis* and certain *Plasmodium sp* DHFR-TS [31].

#### Phospholipid metabolism

Phospholipid metabolism in apicomplexans is well-documented, and highlighted by studies in *B*. *bovis* showing a markedly greater phospholipid composition in infected erythrocytes compared to uninfected erythrocytes [32]. *T*. *equi* has an increased number of choline/ethanolamine kinase genes, even relative to *B*. *bovis*, which has a demonstrated increase in phosphatidylcholine relative to uninfected bovine erythrocytes [33]. Although only a partial set of enzymes for synthesis of glycosylphosphatidylinositol (GPI) anchors were detected, *T*. *equi* has been demonstrated to incorporate GPI anchors on membrane proteins [34]. Approximately 2% of the proteome (132 proteins; Additional file 3) were predicted to contain both required signatures for GPI anchors; however, this prediction should be used with caution as metabolic labeling studies indicate that there are relatively few GPI anchored protein species within infected erythrocytes, with members of the EMA family being the predominantly labeled proteins [34].

##### Transporter families

TransportDB predicts *T*. *equi* to have the most transporters of any hemoparasite genome sequenced to date [26-29], with 142 in total (Additional file 4). The most profound increase is within the ATP-binding cassette (ABC) superfamily of transporters, with *T*. *equi* having 45 members of this family, compared to just 17 in *T*. *parva*, and 9 in *B*. *bovis*. Comparatively, *Plasmodium falciparum* has 16 members of the ABC family, most notably ABCB1 (MDR1), a known mediator of chloroquine and mefloquine resistance [35]. Resistance to chemotherapeutics also occurs in *T*. *equi*, however specific mechanisms of resistance are unknown. Given the high number of ABC transporter family members in *T*. *equi*, including those of the MDR1 type and orthologues of the other known drug transport members ABCC1 and ABCG2, it is reasonable to hypothesize that ABC-mediated transport contributes to chemotherapeutic resistance in *T*. *equi*. Another expanded family is the Type II general secretory pathway, which contains 11 transporters, ~three times the number in the other hemoparasite genomes (Additional file 4). This pathway moves signal peptide containing proteins across the cell membrane. The abundance of signal peptide-containing proteins without predicted transmembrane domains suggests that *T*. *equi* has a large secretome that utilizes this pathway.

As noted earlier, the folate/biopterin family of transporters is increased (at 5), and thus, *T*. *equi* may import additional folate to contribute to the one carbon pool. Finally, *T*. *equi* has 23 transporter genes in the Major Facilitator Superfamily (MFS) about twice as many as other hemoparasites. The MFS transporters include drug efflux systems, organophosphate:phosphate exchangers and oligosaccharide:H^+^ symport permeases and are single-polypeptide secondary carriers capable only of transporting small solutes in response to chemiosmotic ion gradients [36].

### Chromosomal synteny

Figure 1 shows blocks of synteny shared between *T*. *equi* and *B*. *bovis*, *T*. *parva*, or *T*. *annulata*. There are relatively few large regions that do not have syntenic matches in the other piroplasm genomes. Analysis of chromosome 1 demonstrated that these regions contain unique proteins (184), without Pfam hits or other functional assignment. The *T*. *parva* and *T*. *annulata* chromosomes are highly syntenic, and a much more fragmented pattern of synteny is seen between *Theileria* spp. and *B*. *bovis*[29,37]. Large regions of synteny are observed between the *Theileria* spp. and *T*. *equi*, suggesting more recent shared ancestry than for *T*. *equi* and *B*. *bovis*, where the blocks of synteny are more fragmented. *T*. *equi* chromosome 1 appears to be evolutionarily related to *Thelieria spp*. chromosomes 1 and 2. The ancestral *Theileria* chromosome 2 has split to provide elements of *T*. *equi* chromosomes 1 and 2. Approximately 2/3 of each of *T*. *equi* chromosomes 2 and 3 share synteny with *Theileria spp*. chromosomes 1 and 4, respectively. The remaining 1/3 of each of these chromosomes contains more of the *B*. *bovis* specific lineage sequences or unique sequences. With a few exceptions, *T*. *equi* chromosome 4 and is syntenic to both *T*. *parva* and *T*. *annulata* chromosome 3. Notably, the orthologous gene matches extend to the ends of each of the *T*. *equi* chromosomes demonstrating the lack of telomerically located species-specific gene families and repeats seen in other hemoparasites [26,28].

### Paralogous families

TribeMCL placed 2614 proteins in 334 families ranging in size from 2 to 356 members (Additional file 5). Many of the families, including the two largest, have little significant sequence identity, for any two proteins, in pairwise comparisons; however, this result did not change when we used more stringent parameters for clustering. Therefore, we analyzed the families for common functional annotation and show that most members of a given family share a common functional domain or feature (Additional file 5). Exceptions are family 1 and family 17. A few families have high levels of sequence conservation and readily identifiable functional attributes found through Pfam hits. For example, family 11 contains 42 members with hits to Pfam PF00005, a family of ABC transporters. A survey of *T*. *equi* protein families that have at least 20 members revealed that most of these families are comprised of functional attributes similar to those represented in similar scans of *B*. *bovis* and *Theileria* spp. None of the *T*. *equi* families appear to contain a family of known immunodominant antigenically variable genes, nor are any of the families telomerically associated. Notable families are presented below.

#### Family 3

Containing 109 members, this family has ~30% sequence conservation with a repeat gene family in *T*. *annulata* (*Tar*). The *Tar* genes were reported to be analogous to the *Tpr* genes of *T*. *parva*, however Family 3 does not share significant sequence identity with Tpr [27]. Most members of Family 3 encode a protein of ~50 KDa, with no signal peptide. Although the function of these proteins in *Theileria* is unknown, it is thought that these genes in *T*. *parva* are involved in the generation of diversity [28], with the *Tpr* repertoires appearing to be isolate specific [38]. Like *T*. *annulata*, the *T*. *equi* Family 3 genes are distributed throughout all four chromosomes. Expressed Sequence Tags (ESTs) were found for only seven of the Family 3 genes suggesting that only a small number of these genes are expressed at a given time.

#### Equi merozoite antigen (EMA) family

Family 29 contains 10 members related to the best characterized protein in *T*. *equi*, Equi Merozoite Antigen 1 (EMA1). EMA1, a 34 kDa immunodominant protein, is used as the basis of a cELISA diagnostic test for *T*. *equi*, as this highly conserved antigen is recognized by sera from infected animals worldwide [39]. EMA1 has a GPI anchor, a putative erythrocyte-binding domain shared with certain hemotoxins, and surface-exposed epitopes [34]. Immunoprecipitation of *T*. *equi* proteins by serum from a horse challenged twice at a 2-month interval with *T*. *equi* revealed that in addition to EMA1, EMA2 has a GPI anchor and detectable antibody responses were limited to EMA1-3 proteins [34,40]. Although *ema1* was originally described as a single copy gene, genomic analysis has revealed a total of 10 genes in this family. The amino acid identity ranged from 17 to 55% in pairwise comparisons of family members. EMA2 was previously characterized as 50% identical to EMA1, and is also an immunodominant GPI-anchored protein. EMA1 is more highly expressed in blood stages of the parasite relative to EMA2 while EMA2 is more highly expressed in the tick salivary gland during transmission feeding than EMA1 [2]. These two proteins, along with a third member of this family (EMA3) have been shown to interact with the erythrocyte cytoskeleton [40,41]. A single ortholog of these proteins have been identified in *T*. *annulata* (TAMS1) and *T*. *parva* (mMPSA) but not in other Apicomplexa [42,43]. At least one EST was found for each *ema* family member, with the exception of BEWA_028210 and BEWA_047350, demonstrating that transcription in blood stage parasites takes place for most family members.

A core set of 8 *ema* genes are predicted to encode proteins of ~30 kDa, with the remaining two encoding markedly differently sized proteins, one being truncated (BEWA_034050) and the other (BEWA_047350) being much longer and only having similarity in the C-terminal domain (Additional file 6). Interestingly, the truncated gene produces a transcript, based on EST analysis, as long as the other family members, but which encodes multiple stops in the 5’ region preventing formation of a full length protein. Perhaps this family member is in the process of becoming a pseudogene by disruption of the full reading frame, and an indication that the family may be reduced in size over time.

The *ema* genes are distributed across the genome, with no evidence of clustering or telomeric association. There is no evidence that the *ema* genes undergo recombination or dynamic sequence variation, as might be expected if these genes are involved in immune evasion [16,44]. Therefore, despite their immunodominance, their limited number and lack of a structural basis for variation do not fit with the paradigms seen for *P*. *falciparum var* genes (encoding PfEMP1) or *B*. *bovis ves1* genes (encoding VESA1) where evasion of the immune response through emergence of variable surface antigens is a well characterized mechanism of parasite persistence [18,45].

#### FAINT domains

While not a protein family per se, the FAINT domain was originally detected in *T*. *annulata* and *T*. *parva* as a stretch of 70 amino acids and was named “Frequently Associated IN *Theileria*”; subsequently, Pfam04385, the FAINT domain – a domain of unknown function (DUF529) was established and was found to be over represented in proteins predicted to be secreted [27,28]. *T*. *equi* proteins may have multiple FAINT domains and are typically classified into several protein families. *T*. *equi* contains 271 proteins with 560 hits to Pfam04385. FAINT domains are not reported for *B*. *bovis* or *P*. *falciparum*. The FAINT domain containing genes are distributed throughout the *T*. *equi* genome.

### *T*. *equi* does not contain homologs of putative *Theileria* host cell transforming genes

**Figure 2:**
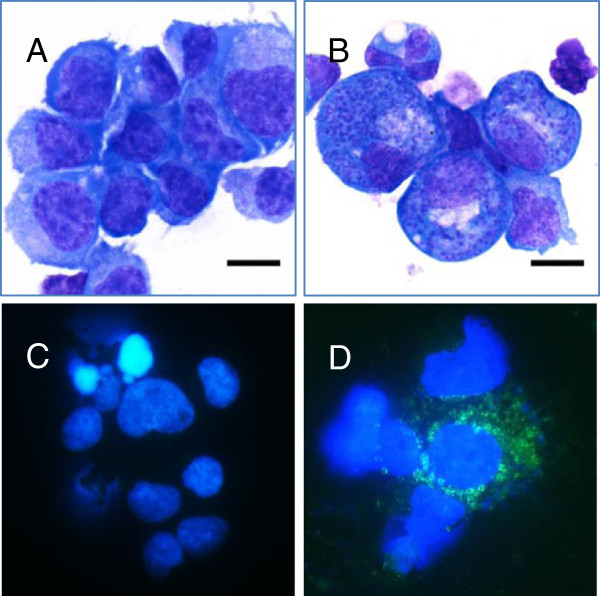
**Analysis of schizont**-**infected PBMC *in vitro.*** The capacity of *T*. *equi* (Florida) to infect PBMC *in vitro* was assessed by light microscopy (**A**, **B**) and IFA (**C**, **D**). Fresh PBMC from adult Arabian horses were cocultured with tick salivary gland homogenates containing *T*. *equi* sporozoites. Infected and uninfected control PBMC were sampled daily for cytospin preparation and Diff-Quick staining [representative photomicrographs of uninfected control cultures (**A**) and *T*. *equi* infected cultures (**B**) on day 9]. Panel B includes three schizont-infected leukocytes, with multiple, oval to round, 1–2 μm diameter, purple nuclei (developing merozoites). To confirm the intracytoplasmic organisms were *T*. *equi*, uninfected control and infected cultures were labeled with antibody specific for equine merozoite antigens 1 and 2 [mAb 36/133.97 (anti-EMA 1/2)]. In the infected culture wells (**D**), intracytoplasmic schizonts and developing merozoites were specifically labeled with anti-EMA 1/2 (secondary goat anti-mouse IgG1 conjugated with FITC-green; Nuclear stain = DAPI). Cells from the uninfected control cultures were not labeled with anti-EMA 1/2 (representative data in panel **C**). Scale bar = 10 μm.

Although host cell transformation has not been reported for *T*. *equi*, it has been reported to invade the lymphocyte and develop to a macroshizont stage [13]. In *T*. *parva* and *T*. *annulata*, the macroschizont life cycle can induce transformation of the infected cell [46]. To assure that the *T*. *equi* isolate used in this study infected equine lymphocytes/PBMCs, equine PBMCs were infected with sporozoites obtained from infected adult male *Rhipicephalus microplus* ticks. To verify infection of equine PBMC cultures, EMA-1 and EMA-2 monoclonal antibodies were tested by immunofluorescence and bound corresponding *T*. *equi* antigens within equine PBMCs (Figure 2). The lack of observed transformation in this close relative of pathogens (*T*. *parva* and *T*. *annulata*) that do transform their host led us to search the *T*. *equi* genome for putative host cell transformation genes to see if these genes were retained in the genome. A set of genes [46] identified as potential manipulators of host cell phenotype in *T*. *annulata* and *T*. *parva* was used to explore the *T*. *equi* genome for orthologous genes, along with the EST expression data. *T*. *parva* and *T*. *annulata* express prohibitin in macro- and microschizonts, while *T*. *equi* expresses the ortholog (BEWA_014000) in erythrocyte-stage parasite ESTs. Prohibitin is reported to be a tumor suppressor that is involved in pathways that lead to immortalization, however, this gene was also identified in *B*. *bovis*, which does not undergo transformation [46]. Similarly, *T*. *equi* has several homologs of cyclophilins, which have been linked to transformation. This gene is found in *B*. *bovis*, *T*. *parva* and *T*. *annulata*, again, limiting the implication of this gene product as a host cell transforming factor. *T*. *equi* does not have a homolog of the *Theileria* schizont AT hook (TashAT) family of proteins. The AT hooks function as nuclear localization signals and DNA binding domains, and may function in cell transformation. Likewise, TashHN (host nucleus) and SuAT1 do not have *T*. *equi* orthologs. TashHN is expressed by *Theileria* macroschizonts that have lost the ability to form merozoites, while SuAT1 is a schizont protein with characteristics of the TashAT family, that can modulate the phenotype of cultured bovine macrophages by altering the expression profile of cytoskeletal proteins in a manner similar to that seen during infection. 

### Vaccine candidates

#### The RAP-1 Family

Members of the rhoptry associated protein-1 (*rap1*) gene family are candidates for vaccine development in *Babesia* spp. Immunization of cattle with purified native RAP-1 of *B*. *bigemina* resulted in partially protective responses, and is one of three antigens in a recombinant vaccine developed in Australia [47]. However, recombinant *B*. *bovis* RAP-1 failed to elicit protective immune responses in vaccine trials. The RAP-1 proteins of *Babesia spp*. are characterized by a signal peptide followed by a cysteine-rich region and several short conserved sequence motifs within the first 300 amino acids. Thus, the N-terminal region of all known *Babesia* RAP-1 proteins contains the “rap-1 domain”. The C-terminal regions are less conserved among *Babesia* species. *B*. *bovis* contains two identical *rap1* genes in tandem and a shorter *rap1* related protein gene (RRP) located ~30 kbp from the *rap1* locus. Three *rap1* genes were identified in *T*. *parva* and four in *T*. *annulata*. The general structure of *rap1* in *Theileria* spp. is different from that in *B*. *bovis*, with the *Theileria* proteins containing more than one rap-1 domain. The *T*. *equi rap1* locus contains two tandemly arranged genes: BEWA_037610 contains two rap-1 domains, and BEWA_037600 contains three rap-1 domains. Thus, the arrangement of rap-1 domains in *T*. *equi* resembles that found in other *Theileria spp*.

#### Apical Membrane Antigen 1

*T*. *equi* Apical Membrane Antigen-1 (AMA-1; BEWA_036830) is a microneme protein with surface exposed epitopes that has orthologs in hemoprotozoan Apicomplexa, as well as *Toxoplasma gondii*[48]. AMA-1 antibody has inhibitory effects on parasite replication *in vitro*, and has been protective in animal models [49-51]. *T*. *equi* and *B*. *bovis* AMA-1 lacks the N-terminal extension found in other *Theileria spp*. AMA-1 has a set of conserved cysteine residues which may contribute to a conserved architecture through disulfide bonds. *Plasmodium* AMA-1 is polymorphic [52], and the degree of polymorphism must be assessed in *T*. *equi* before AMA-1 can be considered as a vaccine candidate for this species.

#### Thrombospondin-related anonymous protein (TRAP)

Conserved hypothetical proteins BEWA_005690 and BEWA_005710 fall into a Cluster of Orthologous Groups (COG) with apicomplexan genes annotated as TRAP, sporozoite surface protein or microneme protein 2 [53]. Although these molecules have been effective immunogens in *Plasmodium*[54], the *T*. *equi* proteins have low sequence similarity and it is unclear whether these would be effective vaccine candidates.

#### Other prophylaxis opportunities suggested by comparative genomics

HAP2 is a protein essential for membrane fusion during zygote formation in *Plasmodium* that has a homolog in numerous species, including *Leishmania* and *T*. *parva*[55]. A positional homolog i.e., a gene with very low sequence identity couched within a highly conserved locus, exists in *B*. *bovis* (BBOV_III006770) and *T*. *equi* (BEWA_7380). This target could provide a novel method of transmission blocking through gene knock out, thus preventing gamete fusion and tick transmission of a modified live vaccine. Membrane Occupation and Recognition Nexus protein (MORN1) seems to be related to cell division in all Apicomplexa studied, and functions in both asexual and sexual reproduction [56]. A common and effective prophylaxis for members of the phylum could come from targeting this molecule. BEWA_033160 is annotated as a MORN repeat domain-containing protein, and falls in to a COG with proteins from *Plasmodium*, *Theileria*, and *B*. *bovis*, as well as *Toxoplasma gondii* and *Cryptosporidium parvum*. Heme Detoxification Protein (HDP) has a role in detoxifying heme that results from parasite metabolism of hemoglobin by polymerizing the heme into hemozoin [57]. BEWA_011450, a putative HDP, is in a COG conserved across apicomplexan organisms. This hypothetical target for prophylaxis would allow toxic levels of heme to accumulate through inactivation of HDP.

##### p67 locus synteny

*T*. *parva* p67 is an abundant sporozoite surface antigen, and along with its homolog from *T*. *annulata* (SPAG-1) has been shown to induce neutralizing antibody, and immunity to theileriosis and has been a target of vaccine development [58]. A positional homolog was identified in *B*. *bovis* (BBOV_IV007750) [29,59] and *T*. *equi* contains a gene at the same location (BEWA_015160). EST data show that BEWA_015160 is transcribed. The prospect that the genes occupying this position in syntenic loci have similar functions during parasite infection but tailored to different hosts makes them interesting candidates for vaccine studies.

##### *The* T. parva *six*

A set of six *T*. *parva* antigens that are the targets of CD8^+^ T lymphocyte responses in immune cattle have been designated Tp1,Tp2, Tp4,Tp5, Tp7 and Tp8 [60]. Tp2 is a surface protein whose homolog is also expressed in *T*. *annulata*. All of these vaccine candidates except hypothetical protein Tp1 have orthologs in *T*. *equi* and have been demonstrated to be transcribed in erythrocyte stages via EST analyses. While the significance of piroplasm stage expression is unclear, these *T*. *equi* proteins may have utility as vaccine components based on observations in *T*. *parva*.

### Phylogenetic analysis of piroplasms

**Figure 3:**
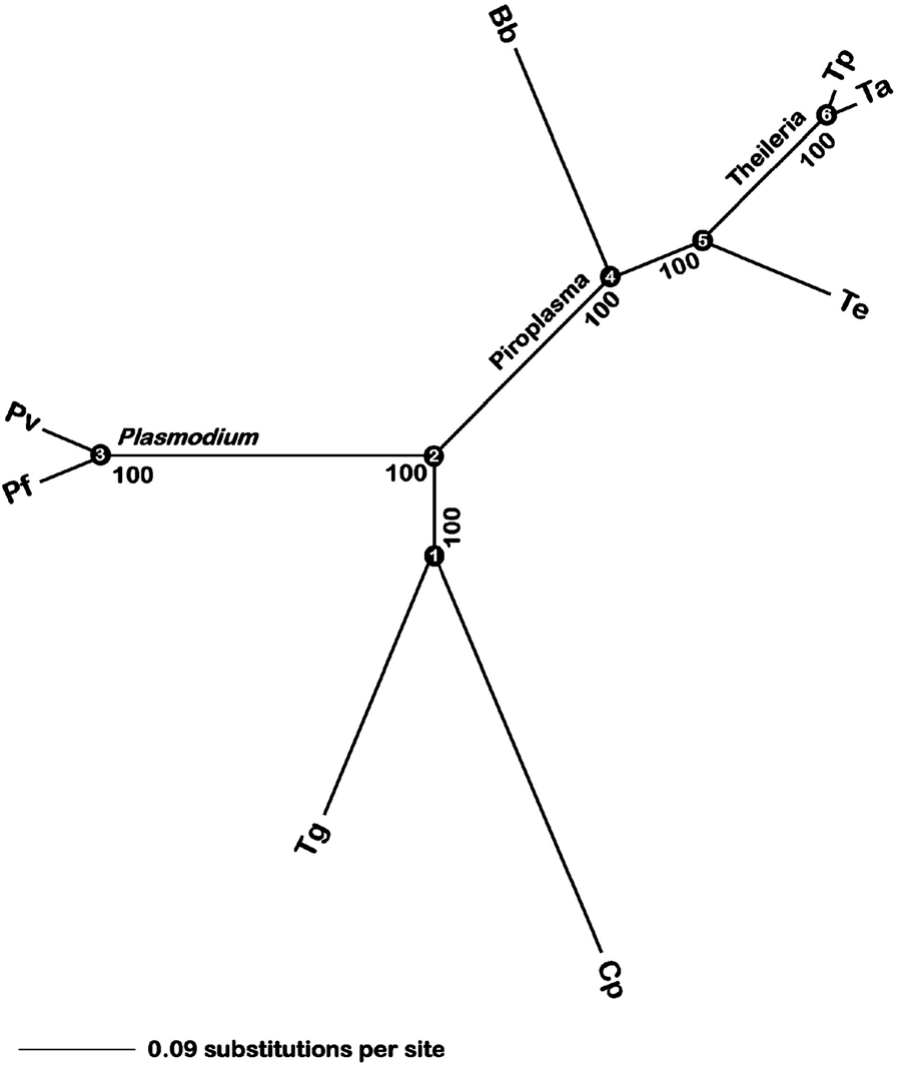
**Phylogenetic tree of sequenced apicomplexans.** Posterior probability distribution tree representing best likelihood score (probability of 1.0) following Bayesian analysis of 150 concatenated polypeptides across eight taxa. Taxa codes are Pf (*Plasmodium falciparum*), Pv (*Plasmodium vivax*), Tg (*Toxoplasma gondii*), Cp (*Cryptosporidium parvum*), Bb (*Babesia bovis*), Te (*Theileria equi*), Tp (*Theileria parva*), Ta (*Theileria annulata*).

Mehlhorn and Schein renamed *Babesia equi* as *T*. *equi* in 1998 due to the description of a lymphocytic stage for this parasite [8]; however, while this classification has been well adopted, it is not borne out by phylogenetic studies. The phylogenetic position of *T*. *equi* was explored using 150 deduced amino acid sequences (Additional file 7) from eight fully sequenced apicomplexan genomes. Results of the Bayesian analysis (Figure 3) support two possible scenarios: the four piroplasm species, *T*. *parva*, *T*. *annulata*, *B*. *bovis* and *T*. *equi*, represent a single genus or alternatively, these species represent three separate genera. Maximum Parsimony (MP) analyses of individual sequences resulted in many different tree topologies; however, the four most commonly recovered topologies (Additional file 8) represent 78% of all trees. Consistent with Bayesian inference, the three most common topologies (Additional file 8, topologies a-c) suggest that *T*. *equi* shares a more recent common ancestor with the two *Theileria* species than with *B*. *bovis*. MP analysis of the concatenated datasets unambiguously recovered this same relationship of the four piroplasm species, and completely corroborated the tree estimated using Bayesian inference. Finally, maximum likelihood analyses of the concatenated datasets using a variety of substitution models (JTT, MTREV, WAG, RTREV, CPREV, VT) consistently yielded the same estimates, which completely corroborate the trees generated by Bayesian inference and MP analyses of the concatenated datasets (Additional file 8). Collectively, robust phylogeny estimation, which utilized three separate optimality criteria and various models of protein evolution, consistently placed *T*. *equi* as a sister taxon to the *Theileria* clade, with all three of these piroplasm species subtended by *B*. *bovis*. 

Previous studies employing 18S rRNA sequences across many piroplasmid taxa also revealed uncertainty in the phylogenetic position of *T*. *equi* and several other piroplasms [61,62]. Our robust phylogenetic analyses, which strongly imply the placement of *T*. *equi* as sister to the *Theileria* clade provide stability to piroplasm systematics. Our goal was to determine if a selection of orthologous polypeptides from completed *Babesia* and *Theileria* genomes could be used to discriminate the appropriate phylogenetic position of *T*. *equi*. However, species sampling was restricted by available complete genome sequences and is too limited to completely resolve this issue. A more appropriate solution may be to place *T*. *equi* in a genus distinct from both *Theileria* and *Babesia*, as has been previously suggested [9]. This option would likely result in the renaming of some related taxa including *Cytauxzoon felis*, which groups as the closest relative of *T*. *equi* in the 18S rRNA study [61]. Importantly, our analysis revealed a set of 50 informative genes that could be analyzed in a broader sampling of Piroplasmida taxa to gain a greater understanding of the evolutionary relationships of the piroplasms (Boxed sequences in Additional file 7).

## Conclusions

Characteristics of genes involved in immune evasion leading to persistence include immunodominance, highly variable domains, and membership in a multigene family. Within the phylum Apicomplexa, *B*. *bovis* contains the 119 member *ves1* gene family which encodes the VESA1, known to vary by gene conversion and participate in immune evasion; and *P*. *falciparum* contains the 59 member *var* gene family encoding PfEMP1, which utilizes several mechanisms to generate antigenic variation resulting in immune evasion [26]. An analogous gene family wasn’t detected in *T*. *equi*, instead, it contains a small family of immunodominant proteins including EMA1, EMA2, EMA3 and seven additional members which have marked variation throughout each gene. While the precise mechanism(s) of antigenic variation provided by the *ema* family isn’t yet known, we hypothesize that EMA family members provide antigenic variation and immune escape through a protein reassortment strategy that generates immune escape variants by creating novel heteromers of EMA on the cell surface. The genome sequence also revealed a 109 member gene family (Family 3); however these proteins have never been detected by immune serum, suggesting that they are not contributing to immune evasion. Genome sequencing allowed an exploration of the phylogenetic position of *T*. *equi* at a depth not previously feasible; demonstrating that this species clusters consistently with, and as a sister lineage to other *Theileria spp*. Genome sequences for closely related piroplasms may allow “fine tuning” of the *T*. *equi* phylogenetic position and appropriate naming of the lineage to which this species belongs. Finally, the genome sequence will provide an invaluable tool for researchers developing methods to control equine piroplasmosis.

## Materials and methods

### Ethics statement

Animal experiments were approved by the Institutional Animal Care and Use Committee at University of Idaho, USA, in accordance with institutional guidelines based on the U.S. National Institutes of Health (NIH) Guide for the Care and Use of Laboratory Animals.

### Parasite culture

#### For sequencing

*Theileria equi* USDA (Florida) stabilate (PPE: 0.64%) generated from infection of a spleen intact horse (H2) was inoculated intravenously into a splenectomized horse (H047). When the parasitemia reached 16% as determined by Giemsa stained blood smear examination, blood was collected into a flask containing glass beads for defibrination. The blood was washed three times with HBSS/10mM EDTA to remove the white blood cells (WBC). Erythrocytes were purified using histopaque 1077 following the manufacturer’s protocol. Microscopic examination determined WBC contamination to be ≪5 WBC per 1 ul of blood. *T*. *equi* genomic DNA was extracted from the purified erythrocytes using components of the Gentra Puregene DNA Purification System (Kit D-5000).

#### *T*. *equi* sporozoite infection of PBMC *in vitro*:

To obtain the sporozoites need for *in vitro* PBMC infection, one thousand adult male *Rhipicephalus microplus* ticks were acquisition fed on a *T*. *equi* (Florida strain) merozoite stabilate-infected adult horse during ascending parasitemia for eight days (acquisition feeding). Following acquisition feeding, fed ticks were incubated for two days at 15°C with 94% relative humidity and a 12 hr photo period, and subsequently transmission fed on a naïve horse to induce final maturation of sporozoites into their infectious stage. On day seven of the transmission feeding, 240 live adult males were recovered and salivary gland pairs were dissected aseptically, and sporozoite extracts prepared using established methods. Briefly, collected salivary glands were washed in 0.5 ml of complete media (RPMI 1640 supplemented with 10% heat inactivated fetal bovine serum, 200 IU/ml benzyl penicillin, 200 ug/ml streptomycin sulphate, 50 μg/ml gentamycin and 5 × 10^-5^ M 2-mercaptoethanol) and crushed in a glass homogenizer to release sporozoites. The homogenate was then centrifuged at 300 g for 5 min and the supernatant containing sporozoites was collected and adjusted to 10 tick equivalents/ml. One ml of the sporozoite extract was inoculated into each of ten wells of a 24-well plate containing 2 × 10^6^ PBMC from an uninfected horse. Starting on day nine post-inoculation, cell culture aliquots were cytospun and examined microscopically for infection following Diff-Quick staining.

### Sequencing

DNA from *T*. *equi* USDA (Florida) strain was used to construct a small and large insert plasmid library as well as a bacterial artificial chromosome library. A total of 100,835 high quality sequence reads (822 bp average read length) were generated and assembled using the Celera Assembler ( http://sourceforge.net/projects/wgs-assembler/), resulting in 119 scaffolds consisting of 495 contigs. A BAC library was end sequenced to generate an additional 3023 reads which were used to confirm the assembly and for targeted sequencing in the closure phase. Gaps were closed by a combination of primer walking and transposon based or shotgun sequencing of medium insert clones, BAC clones or PCR products. The genome sequence has been deposited in GenBank under accession number ACOU00000000. A cDNA library was constructed using cultured infected erythrocytes (Florida strain) using the Creator SMART cDNA library construction kit (Clontech). cDNA in the size range of 0.3 -2kb was normalized using the Trimmer normalization kit (Evrogen). 7629 sequences (GenBank Accession #s HS032712 - HS040340) were generated and assembled into 2355 contigs using PASA [63].

### Genome annotation

Genes encoding tRNA’s were identified using tRNA scan-SE [64]. Gene models were predicted using the ab initio gene finding programs GeneZilla, GlimmerHMM [65], Phat [66] and Snap [67] that used 395 partial and full length high confidence genes in the training set. The training data was manually constructed and inspected for its alignment against highly conserved protein sequences using the AAT package [68] and PASA [63] to align the ESTs to the genomic sequence with a stringent criteria of 95% identity over 90% length using gmap [69]. Data were combined and consensus gene models were derived using Evidence Modeler EVM [70]. The consensus gene models were manually checked for obvious errors. Such models were corrected using a Java based tool called Neomorphic Annotation Station [71].

Functional annotation was as for *B*. *bovis*[29]. In addition, EC numbers were assigned in an automated fashion using PRIAM [72] and metabolic pathways constructed using SRI’s pathway tools [73]. TransportDB is a relational database that was used to determine transporter complement [74]. TribeMCL [75] was used with default parameters to construct a database of genes that are part of paralogous families of proteins represented in the genome. The method is a sequence similarity matrix-based Markov clustering method.

### Comparative genome analysis

Sybil ( http://sybil.sourceforge.net) was used to create an all-versus-all BLASTP search using the proteomes of *T*. *equi*, *B*. *bovis*, *T*. *parva*, *T*.*annulata*, *P*. *falciparum*, *P*. *vivax*, *C*. *parvum and T*. *gondii*. These outputs were subjected to Jaccard clustering, placing proteins into distinct clusters for each proteome. Clusters from different proteomes were linked based on best bidirectional BLASTP hits between them to provide Jf-COGs. A minimum block size of five with one gap was allowed in the analyses.

### Immunofluorescence assay

To verify PBMC cultures were infected with *T*. *equi*, cytospin preparations were made for immunofluorescence antibody microscopy on day nine after cell culture inoculation. After drying, cytospin preparations were fixed in 1% formalin for 2 min and primarily labeled with *T*. *equi* EMA-1 and EMA-2-specific monoclonal antibody 36/133.97. Bound antibody was detected using fluorescein-conjugated goat anti-mouse IgG1 and subsequently visualized by fluorescence microscopy.

### Phylogenetic analysis

The phylogenetic position of *T*. *equi* relative to other piroplasm species with published genomes, namely *T*. *parva*, *T*. *annulata*, and *B*. *bovis* was determined based on 150 polypeptide sequences, and rooted with four other apicomplexan taxa: *P*. *vivax*, *P*. *falciparum*, *T*. *gondii*, and *C*. *parvum*. Polypeptides were selected from single copy genes identified in all eight species. COGs (clusters of orthologous genes) were defined using an in-house comparative pipeline, which starts with BLASTP among protein sequences within and across species. We used the BLOSUM62 matrix with expected value 10^-5^. Jaccard clustering was then performed twice, once to form within-species clusters of paralogous genes and a second time to derive a set of multi-species COGs. In the first case, we used an 80% identity cutoff over a minimum of 70% of the length of the smallest protein and a link score of 0.6. In the second, we set the identity cutoff at 50% over >70% length, and Jaccard coefficient cutoff of 0 for edge pruning. In order to minimize ambiguous amino acid homology assignments that can occur when indels are present, peptides were chosen that have the smallest protein size variation across all species (Additional file 7 for list). Protein sequences within a COG were aligned with ClustalW using default parameters [76].

Three methods were used to reconstruct the phylogenetic relationships among the sequences: i) maximum parsimony (MP) with exhaustive tree search, and otherwise default parameters as implemented in PAUP v4.0b10 [77], ii) Bayesian inference (BI) implemented in MrBayes v3.1.2 [78], and iii). maximum likelihood (ML) estimation as implemented in RAxML v.7.2.8 [79]. Initially, the individual protein datasets were analyzed with maximum parsimony, with branch support assessed with 1000 bootstrap replicates. The 150 datasets were subsequently concatenated and again analyzed using MP with exhaustive tree search and similar bootstrapping procedure. For BI of the concatenated datasets, the amino acid transition matrix was set to a mixture of models with fixed rate matrices (Poisson, Jones, Dayhoff, Mtrev, Mtmam, Wag, Rtrev, Cprev, Vt and Blosum) of equal prior probabilities, and otherwise default parameters. Four runs of MrBayes were conducted, each with 4 chains. Two of those runs ran for 567,000 generations and the other two ran for 2,365,000 generations. Convergence was achieved (Potential Scale Reduction Factor, PSRF=1.00) for all model parameters estimated, including tree length (mean-1.79), the amino acid model (Wag, with posterior probability=1.00), and the tree topology. ML analyses of the concatenated datasets all implemented a gamma model of rate heterogeneity with estimation of the proportion of invariable sites. Six separate amino acid substitution models (JTT, MTREV, WAG, RTREV, CPREV, VT) were utilized. Branch support was assessed with 1000 bootstrap pseudoreplications.

Note: Two additional genome sequences of interest were published while this article was in review. *T*. *orientalis* does not transform it’s host cell and has been compared to *T*. *parva* and *T*. *annulata* to identify putative mediators of leukocyte transformation [80]. *B*. *microti* phylogeny was analyzed using 316 single copy genes and found to clade separately to *T*. *parva*, *T*. *annulata* and *B*. *bovis*[81].

## Abbreviations

ABC: ATP-binding cassette; AMA: Apical Membrane Antigen; COG: Cluster of Orthologous Groups; DHFR-TS: Dihydrofolate reductase-thymidylate synthase; EMA: Equi Merozoite Antigens; EST: Expressed sequence tag; FAINT: Frequently Associated IN *Theileria*; GPI: Glycosylphosphatidylinositol; MFS: Major Facilitator Superfamily; MP: Maximum Parsimony; mt: Mitochondrial; MORN: Membrane Occupation and Recognition Nexus; PBMC: Peripheral blood mononuclear cells; *rap*: Rhoptry associated protein; TCA: Tricarboxylic acid cycle; VESA: Variant erythrocyte surface antigen; WBC: White blood cells.

## Competing interests

The authors declare that they have no competing interests.

## Authors’ contributions

LSK, MT, DRH, AD, JJG, JCS, VMN, RHM, DPK and KAB conceived and designed the experiments. LSK, MT, DRH, JDR, EC, AD, JJG, MGS, AOTL, HER, JCS, CES, and MWU performed the experiments. LSK, MT, DRH, JDR, EC, AD, JJG, MGS, AOTL, HER, JCS, CES, MWU, RHM, DPK, and KAB analyzed the data. LSK, DRH, DPK, and KAB wrote the paper. All authors read and approved the final manuscript.

## Supplementary Material

Additional file 1 **Figure.** Depiction of the *T*. *equi* apicoplast genome gene arrangement, showing unidirectional coding of genes. Known enzymes shown in red, ribosomal proteins in green, rRNA sequences in yellow, and groupings of tRNA molecules in blue. Location of conserved hypothetical (gray), and hypothetical (black) protein-encoding genes are shown by arrows or bars. Members of the three expanded gene families are marked with either “*”, “¡” or “^” to indicate similar genes. The molecule is depicted as linear, though not experimentally demonstrated to be either circular or linear. Click here for file

Additional file 2 **Table.** Nuclear encoded genes potentially targeted to the apicoplast. Click here for file

Additional file 3 **Table.** GPI anchored proteins predicted by GPI-SOM. Click here for file

Additional file 4 **Table.** Transporter comparison *B.bovis*, *T*. *equi*, and *T.parva.* Click here for file

Additional file 5 **Table.** 30 largest protein families. Click here for file

Additional file 6 **Figure.** Alignment of EMA family sequences. Residues highlighted in yellow are conserved among all family members, and those in blue conserved among the majority of family members. Dashes represent gaps introduced to accommodate non-conserved stretches of sequence. Click here for file

Additional file 7 **Table.** Proteins used in phylogenetic analysis. Click here for file

Additional file 8 **Figure.** Phylogenetic trees. A: Most frequently recovered trees from maximum parsimony analysis of 150 polypeptides conserved among eight taxa, showing the number of times tree recovered out of 210 total topologies. Value above the line represents the number times that branch was recovered out of total times whole topology was recovered , and value below the line is percentage bootstrap support for that branch out of 1000 replicates. The bootstrap support for the individual MP trees was calculated for each individual dataset, and averages across all of the individual trees are presented. B: Single most parsimonious tree estimated from the concatenated dataset of the 150 polypeptides. Taxon codes are Cp: *Cryptosporidium parvum*, Tg: *Toxoplasma gondii*, Pf: *Plasmodium falciparum*, Pv: *Plasmodium vivax*, Bb: *Babesia bovis*, Te: *Thieleria equi*, Ta: *Theileria annulata*, Tp: *Theileria parva*. C: Trees estimated with maximum likelihood using six different models of amino acid substitution. Branch support was assessed with 1000 bootstrap pseudoreplications. Click here for file
