# Supplementary figures and images for: Comparative genomic analysis and phylogenetic position of Theileria equi
Source: BMC Genomics. 2012 Nov 9;13:603. doi: 10.1186/1471-2164-13-603 (PMC3505731; doi:10.1186/1471-2164-13-603)

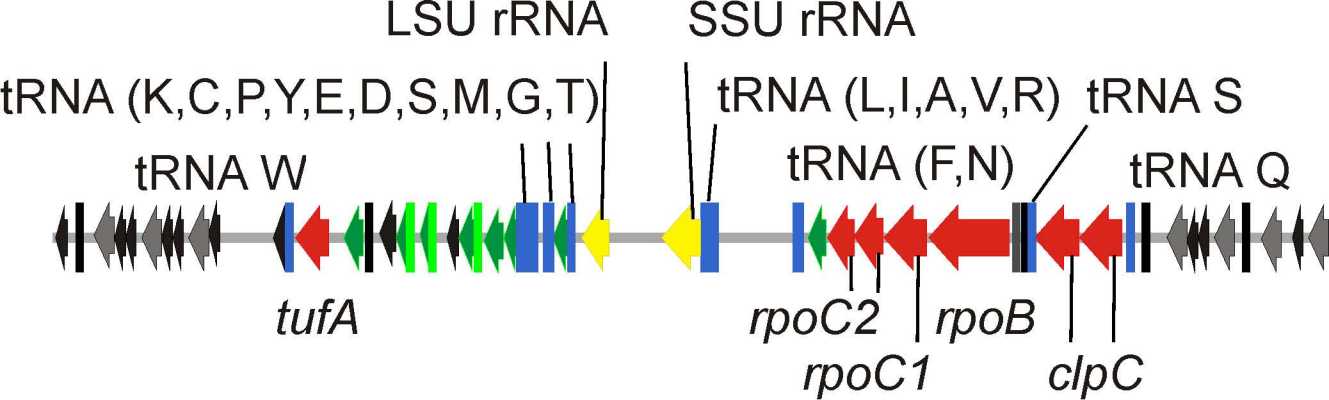

*T. equi* Apicoplast

47,880 bp

Supplement: Additional file 1 — Figure. Depiction of the T. equi apicoplast genome gene arrangement, showing unidirectional coding of genes. Known enzymes shown in red, ribosomal proteins in green, rRNA sequences in yellow, and groupings of tRNA molecules in blue. Location of conserved hypothetical (gray), and hypothetical (black) protein-encoding genes are shown by arrows or bars. Members of the three expanded gene families are marked with either “*”, “¡” or “^” to indicate similar genes. The molecule is depicted as linear, though not experimentally demonstrated to be either circular or linear. [file 1471-2164-13-603-S1.pdf]

**A**

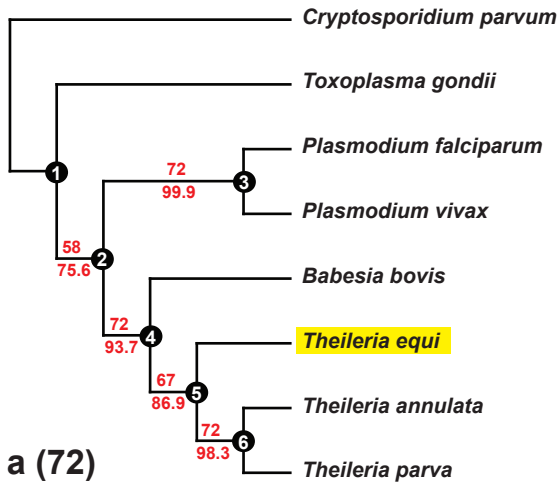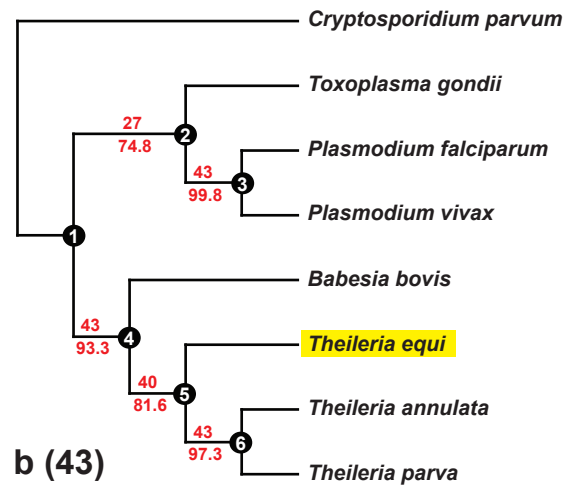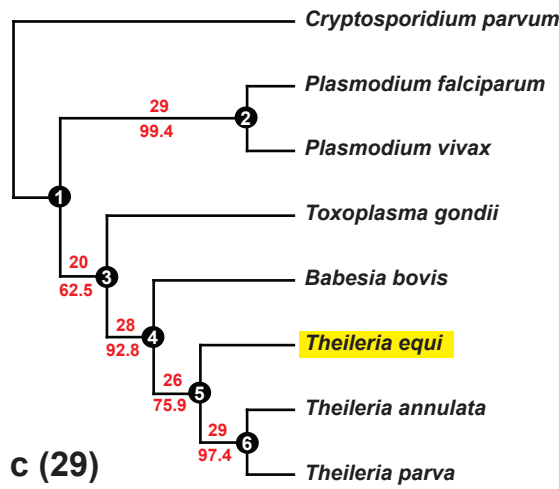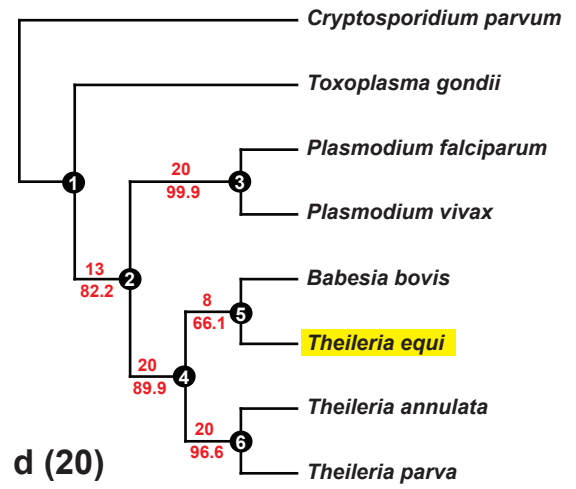

**B**

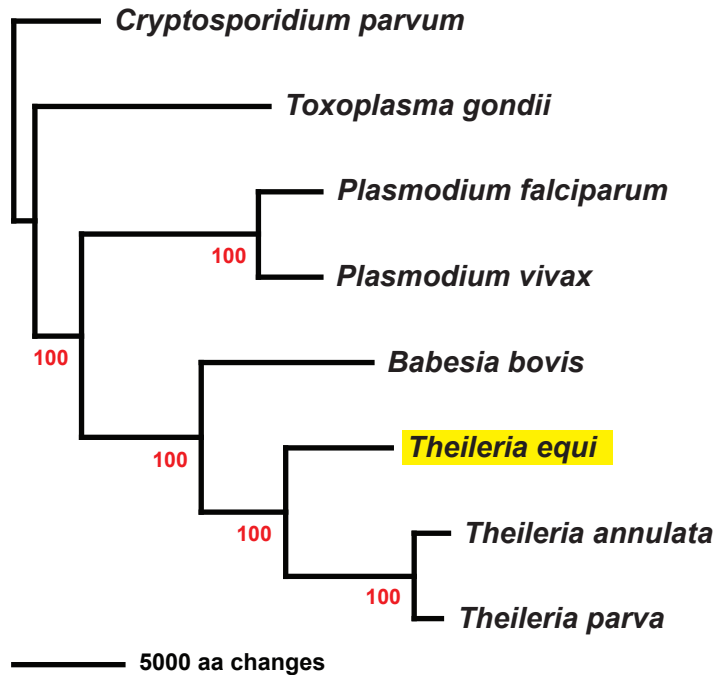

C

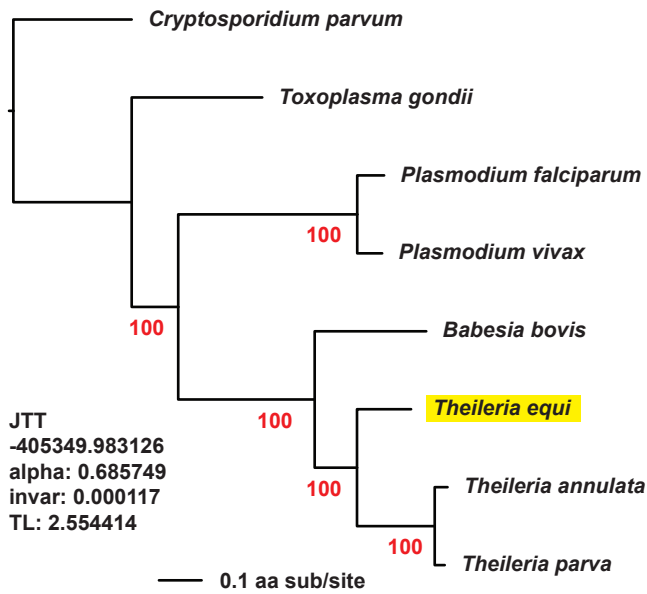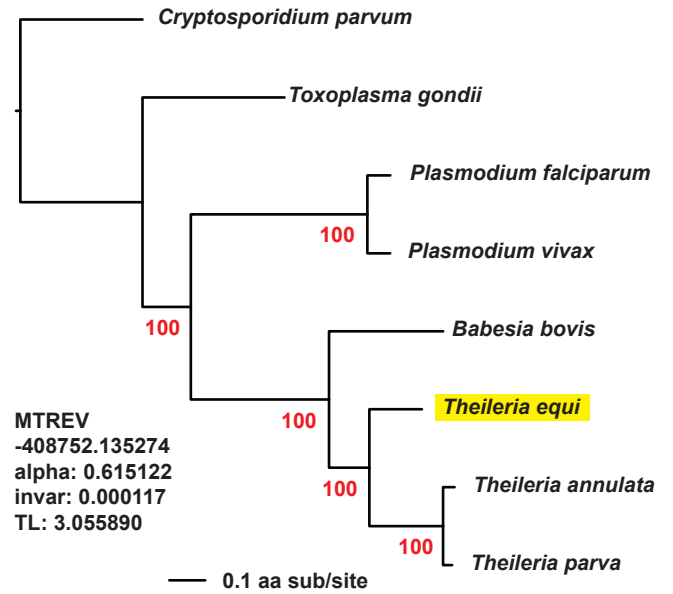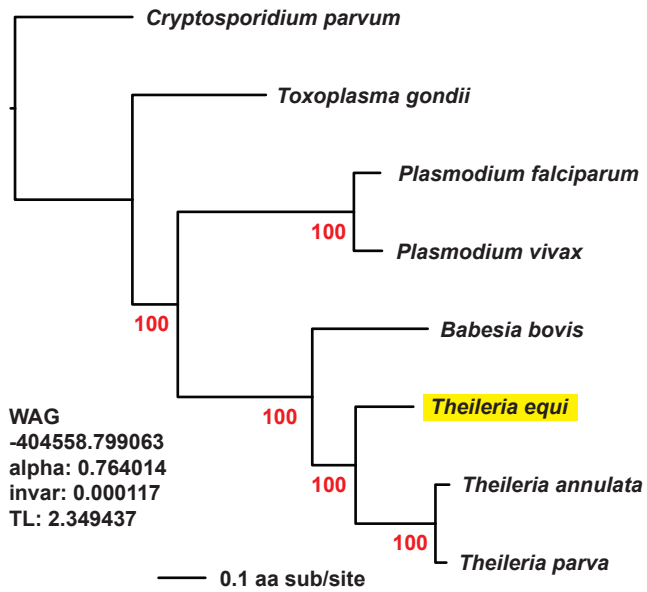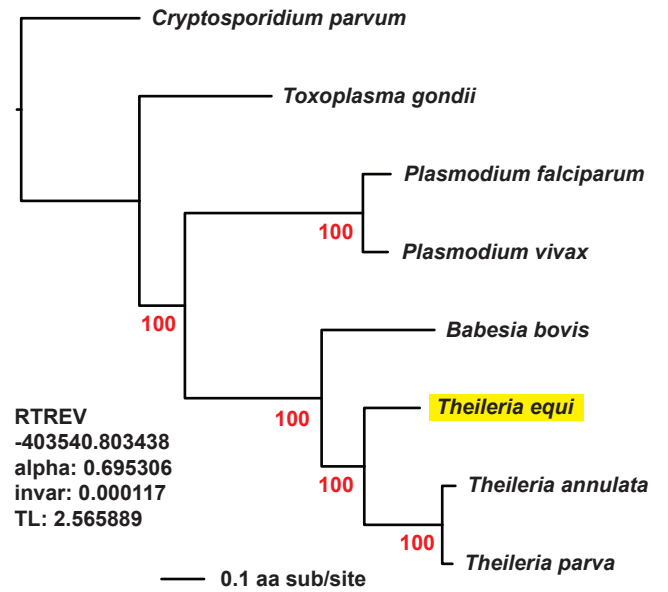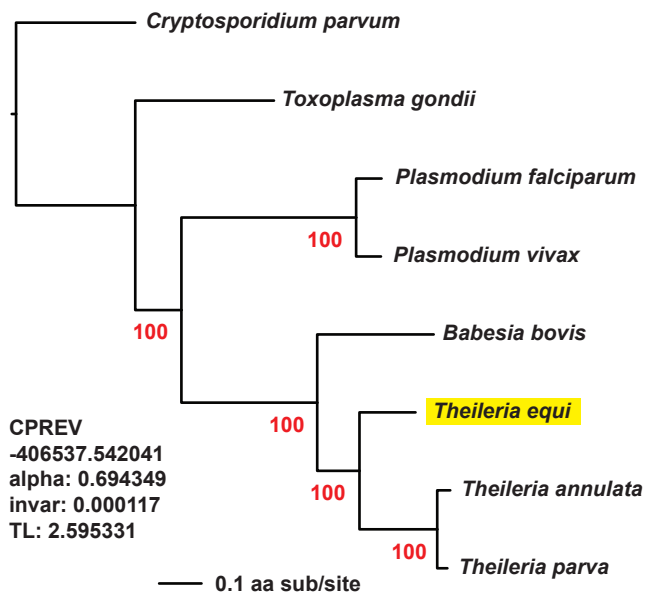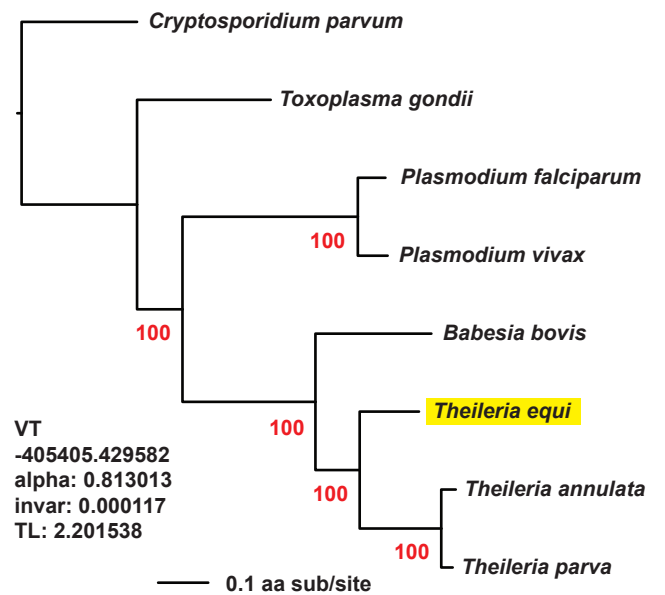

Supplement: Additional file 8 — Figure. Phylogenetic trees. A: Most frequently recovered trees from maximum parsimony analysis of 150 polypeptides conserved among eight taxa, showing the number of times tree recovered out of 210 total topologies. Value above the line represents the number times that branch was recovered out of total times whole topology was recovered , and value below the line is percentage bootstrap support for that branch out of 1000 replicates. The bootstrap support for the individual MP trees was calculated for each individual dataset, and averages across all of the individual trees are presented. B: Single most parsimonious tree estimated from the concatenated dataset of the 150 polypeptides. Taxon codes are Cp: Cryptosporidium parvum, Tg: Toxoplasma gondii, Pf: Plasmodium falciparum, Pv: Plasmodium vivax, Bb: Babesia bovis, Te: Thieleria equi, Ta: Theileria annulata, Tp: Theileria parva. C: Trees estimated with maximum likelihood using six different models of amino acid substitution. Branch support was assessed with 1000 bootstrap pseudoreplications. [file 1471-2164-13-603-S8.pdf]
