# Supplementary material for: Comparative genomic analysis and phylogenetic position of Theileria equi
Source: BMC Genomics. 2012 Nov 9;13:603. doi: 10.1186/1471-2164-13-603 (PMC3505731; doi:10.1186/1471-2164-13-603)
Supplement: Additional file 2 — Table. Nuclear encoded genes potentially targeted to the apicoplast. [file 1471-2164-13-603-S2.pdf]

**Table: Nuclear encoded genes potentially targeted to the apicoplast**

| Gene identifier | Functional name | SP[a] | Pathway[b] | PlasmoAP[c] | ApicoAP[d] |
|---|---|---|---|---|---|
| BEWA_045000 | farnesyl pyrophosphate synthetase, putative | - | + | - | - |
| BEWA_051900 | 4-hydroxybenzoate octaprenyltransferase | - | + | + | - |
| BEWA_053100 | LytB protein | + | + | - | + |
| BEWA_053830 | 1-deoxy-D-xylulose 5-phosphate synthase family protein | - | + | - | + |
| BEWA_016520 | 2-C-methyl-D-erythritol 4-phosphate cytidylyltransferase | + | + | - | + |
| BEWA_017980 | 1-deoxy-D-xylulose 5-phosphate reductoisomerase family protein | + | + | - | + |
| BEWA_024900 | 4-hydroxy-3-methylbut-2-en-1-yl diphosphate synthase (gcpE protein) | + | + | - | + |
| BEWA_014460 | 2-C-methyl-D-erythritol 2,4-cyclodiphosphate synthase, putative | + | + | - | + |
| BEWA_021220 | *tRNA pseudouridine synthase B, putative* | + | - | + | + |
| BEWA_017600 | conserved hypothetical protein | - | - | + | - |
| BEWA_033110 | MAC/perforin domain containing protein | + | - | + | - |
| BEWA_006100 | tyrosyl-tRNA synthetase, putative | - | - | + | - |
| BEWA_034310 | 50S ribosomal protein L33 | - | - | + | + |
| BEWA_001330 | tRNA modification GTPase TrmE , putative | + | - | - | + |
| BEWA_020470 | apurinic endonuclease (APN1) family protein | - | - | + | - |
| BEWA_033610 | S-adenosyl methyltransferase, putative | + | - | - | + |
| BEWA_051550 | Der1-like family, putative | + | - | - | + |
| BEWA_047980 | methionyl-tRNA synthetase, putative | - | - | + | - |
| BEWA_045740 | signal peptide containing protein | + | - | + | - |
| BEWA_046200 | signal peptide containing protein | + | - | + | - |
| BEWA_046980 | signal peptide containing protein | + | - | + | - |
| BEWA_048900 | signal peptide containing protein | + | - | + | - |
| BEWA_052050 | conserved hypothetical protein | + | - | + | + |
| BEWA_000400 | hypothetical protein | + | - | + | - |
| BEWA_001070 | hypothetical protein | + | - | + | - |
| BEWA_001870 | signal peptide containing protein | + | - | + | - |
| BEWA_003060 | signal peptide containing protein | + | - | + | - |
| BEWA_005200 | elongation factor ts, putative | + | - | + | + |
| BEWA_006040 | conserved hypothetical protein | + | - | + | - |
| BEWA_007250 | hypothetical protein | + | - | + | + |
| BEWA_009480 | DnaJ domain containing protein | + | - | + | - |
| BEWA_010380 | conserved hypothetical protein | + | - | + | + |
| BEWA_011990 | signal peptide containing protein | + | - | + | + |
| BEWA_014290 | conserved hypothetical protein | + | - | + | + |
| BEWA_014980 | conserved hypothetical protein | + | - | + | + |
| BEWA_015950 | hypothetical protein | + | - | + | + |
| BEWA_016540 | cathepsin E, putative | + | - | + | - |
| BEWA_016810 | signal peptide containing protein | + | - | + | + |
| BEWA_018700 | ubiquitin family member protein | + | - | + | + |

| | | | | | |
|---|---|---|---|---|---|
| BEWA_020210 | GroES chaperonin family member protein | + | - | + | + |
| BEWA_020440 | ubiquitinactivating enzyme E1, putative | + | - | + | + |
| BEWA_020490 | conserved hypothetical protein | + | - | + | - |
| BEWA_021270 | conserved hypothetical protein | + | - | + | + |
| BEWA_023800 | membrane protein, putative | + | - | + | + |
| BEWA_024510 | 50S ribosomal protein L17e, putative | + | - | + | + |
| BEWA_028400 | signal peptide containing protein | + | - | + | - |
| BEWA_028650 | hypothetical protein | + | - | + | + |
| BEWA_030550 | ribosomal protein L35 family member protein | + | - | + | + |
| BEWA_030690 | signal peptide containing protein | + | - | + | - |
| BEWA_031340 | signal peptide containing protein | + | - | + | - |
| BEWA_031440 | hypothetical protein | + | - | + | - |
| BEWA_031930 | signal peptide containing protein | + | - | + | - |
| BEWA_032530 | 40S ribosomal protein S11, putative | + | - | + | + |
| BEWA_033440 | signal peptide containing protein | + | - | + | + |
| BEWA_033570 | conserved hypothetical protein | + | - | + | + |
| BEWA_034480 | tRNAdihydrouridine synthase, putative | + | - | + | + |
| BEWA_034580 | signal peptide containing protein | + | - | + | + |
| BEWA_038170 | signal peptide containing protein | + | - | + | + |
| BEWA_038950 | hypothetical protein | + | - | + | - |
| BEWA_040440 | signal peptide containing protein | + | - | + | + |
| BEWA_041450 | ABC transporter, ATPbinding protein domain containing protein | + | - | + | - |
| BEWA_042770 | methioninetRNA synthetase, putative | + | - | + | + |
| BEWA_043320 | signal peptide containing protein | + | - | + | + |
| BEWA_024880 | 1-acyl-sn-glycerol-3-phosphate acyltransferase, putative | + | - | - | + |
| BEWA_011110 | 30S ribosomal protein S10, putative | + | - | - | + |
| BEWA_017280 | 50S ribosomal protein L15, putative | + | - | - | + |
| BEWA_017270 | 50S ribosomal protein L17, putative | + | - | - | + |
| BEWA_043470 | 50S ribosomal protein L1p, putative | + | - | - | + |
| BEWA_011860 | 50S ribosomal protein L3, putative | + | - | - | + |
| BEWA_023010 | 5'-3' exonuclease, putative | + | - | - | + |
| BEWA_031270 | 60S ribosomal protein L10, putative | + | - | - | + |
| BEWA_024760 | 60S ribosomal protein L28, putative | + | - | - | + |
| BEWA_026070 | ABC transporter, ATP-binding protein family member protein | + | - | - | + |
| BEWA_017820 | actin, putative | + | - | - | + |
| BEWA_007700 | ADP-ribosylation factor family member protein | + | - | - | + |
| BEWA_029100 | aspartate carbamoyltransferase, putative | + | - | - | + |
| BEWA_010630 | ATP-dependent Clp protease ATP-binding subunit, putative | + | - | - | + |
| BEWA_042560 | ATP-dependent Clp protease proteolytic subunit, putative | + | - | - | + |
| BEWA_014020 | BT1 folate/biopterin transporter family protein | + | - | - | + |
| BEWA_012280 | cell division cycle protein 48, putative | + | - | - | + |

| | | | | | |
|---|---|---|---|---|---|
| BEWA_020900 | chaperone protein DnaJ, putative | + | - | - | + |
| BEWA_050490 | chaperonin 60 kDa, putative | + | - | - | + |
| BEWA_047350 | conserved hypothetical protein | + | - | - | + |
| BEWA_047410 | conserved hypothetical protein | + | - | - | + |
| BEWA_048580 | conserved hypothetical protein | + | - | - | + |
| BEWA_048970 | conserved hypothetical protein | + | - | - | + |
| BEWA_049030 | conserved hypothetical protein | + | - | - | + |
| BEWA_050370 | conserved hypothetical protein | + | - | - | + |
| BEWA_051110 | conserved hypothetical protein | + | - | - | + |
| BEWA_051580 | conserved hypothetical protein | + | - | - | + |
| BEWA_051950 | conserved hypothetical protein | + | - | - | + |
| BEWA_052240 | conserved hypothetical protein | + | - | - | + |
| BEWA_053110 | conserved hypothetical protein | + | - | - | + |
| BEWA_053270 | conserved hypothetical protein | + | - | - | + |
| BEWA_053420 | conserved hypothetical protein | + | - | - | + |
| BEWA_054200 | conserved hypothetical protein | + | - | - | + |
| BEWA_054640 | conserved hypothetical protein | + | - | - | + |
| BEWA_001080 | conserved hypothetical protein | + | - | - | + |
| BEWA_004280 | conserved hypothetical protein | + | - | - | + |
| BEWA_004550 | conserved hypothetical protein | + | - | - | + |
| BEWA_005390 | conserved hypothetical protein | + | - | - | + |
| BEWA_005470 | conserved hypothetical protein | + | - | - | + |
| BEWA_005690 | conserved hypothetical protein | + | - | - | + |
| BEWA_006630 | conserved hypothetical protein | + | - | - | + |
| BEWA_006760 | conserved hypothetical protein | + | - | - | + |
| BEWA_007380 | conserved hypothetical protein | + | - | - | + |
| BEWA_007450 | conserved hypothetical protein | + | - | - | + |
| BEWA_007970 | conserved hypothetical protein | + | - | - | + |
| BEWA_008040 | conserved hypothetical protein | + | - | - | + |
| BEWA_008120 | conserved hypothetical protein | + | - | - | + |
| BEWA_009860 | conserved hypothetical protein | + | - | - | + |
| BEWA_010320 | conserved hypothetical protein | + | - | - | + |
| BEWA_012210 | conserved hypothetical protein | + | - | - | + |
| BEWA_012720 | conserved hypothetical protein | + | - | - | + |
| BEWA_012960 | conserved hypothetical protein | + | - | - | + |
| BEWA_014720 | conserved hypothetical protein | + | - | - | + |
| BEWA_014840 | conserved hypothetical protein | + | - | - | + |
| BEWA_015080 | conserved hypothetical protein | + | - | - | + |
| BEWA_015120 | conserved hypothetical protein | + | - | - | + |
| BEWA_015330 | conserved hypothetical protein | + | - | - | + |
| BEWA_015350 | conserved hypothetical protein | + | - | - | + |

| | | | | | |
|---|---|---|---|---|---|
| BEWA_016040 | conserved hypothetical protein | + | - | - | + |
| BEWA_016530 | conserved hypothetical protein | + | - | - | + |
| BEWA_017230 | conserved hypothetical protein | + | - | - | + |
| BEWA_019400 | conserved hypothetical protein | + | - | - | + |
| BEWA_019720 | conserved hypothetical protein | + | - | - | + |
| BEWA_019760 | conserved hypothetical protein | + | - | - | + |
| BEWA_019770 | conserved hypothetical protein | + | - | - | + |
| BEWA_020110 | conserved hypothetical protein | + | - | - | + |
| BEWA_020130 | conserved hypothetical protein | + | - | - | + |
| BEWA_020400 | conserved hypothetical protein | + | - | - | + |
| BEWA_022270 | conserved hypothetical protein | + | - | - | + |
| BEWA_022440 | conserved hypothetical protein | + | - | - | + |
| BEWA_023520 | conserved hypothetical protein | + | - | - | + |
| BEWA_023850 | conserved hypothetical protein | + | - | - | + |
| BEWA_024070 | conserved hypothetical protein | + | - | - | + |
| BEWA_024360 | conserved hypothetical protein | + | - | - | + |
| BEWA_024580 | conserved hypothetical protein | + | - | - | + |
| BEWA_024630 | conserved hypothetical protein | + | - | - | + |
| BEWA_024820 | conserved hypothetical protein | + | - | - | + |
| BEWA_024830 | conserved hypothetical protein | + | - | - | + |
| BEWA_024840 | conserved hypothetical protein | + | - | - | + |
| BEWA_025000 | conserved hypothetical protein | + | - | - | + |
| BEWA_025940 | conserved hypothetical protein | + | - | - | + |
| BEWA_027610 | conserved hypothetical protein | + | - | - | + |
| BEWA_028810 | conserved hypothetical protein | + | - | - | + |
| BEWA_028970 | conserved hypothetical protein | + | - | - | + |
| BEWA_030310 | conserved hypothetical protein | + | - | - | + |
| BEWA_030930 | conserved hypothetical protein | + | - | - | + |
| BEWA_031970 | conserved hypothetical protein | + | - | - | + |
| BEWA_032010 | conserved hypothetical protein | + | - | - | + |
| BEWA_032430 | conserved hypothetical protein | + | - | - | + |
| BEWA_032650 | conserved hypothetical protein | + | - | - | + |
| BEWA_032970 | conserved hypothetical protein | + | - | - | + |
| BEWA_033340 | conserved hypothetical protein | + | - | - | + |
| BEWA_033780 | conserved hypothetical protein | + | - | - | + |
| BEWA_034240 | conserved hypothetical protein | + | - | - | + |
| BEWA_034270 | conserved hypothetical protein | + | - | - | + |
| BEWA_034610 | conserved hypothetical protein | + | - | - | + |
| BEWA_035080 | conserved hypothetical protein | + | - | - | + |
| BEWA_035950 | conserved hypothetical protein | + | - | - | + |
| BEWA_036090 | conserved hypothetical protein | + | - | - | + |

| | | | | | |
|---|---|---|---|---|---|
| BEWA_036720 | conserved hypothetical protein | + | - | - | + |
| BEWA_036740 | conserved hypothetical protein | + | - | - | + |
| BEWA_037940 | conserved hypothetical protein | + | - | - | + |
| BEWA_038180 | conserved hypothetical protein | + | - | - | + |
| BEWA_039070 | conserved hypothetical protein | + | - | - | + |
| BEWA_039500 | conserved hypothetical protein | + | - | - | + |
| BEWA_039620 | conserved hypothetical protein | + | - | - | + |
| BEWA_039890 | conserved hypothetical protein | + | - | - | + |
| BEWA_040170 | conserved hypothetical protein | + | - | - | + |
| BEWA_041300 | conserved hypothetical protein | + | - | - | + |
| BEWA_042150 | conserved hypothetical protein | + | - | - | + |
| BEWA_042370 | conserved hypothetical protein | + | - | - | + |
| BEWA_042380 | conserved hypothetical protein | + | - | - | + |
| BEWA_043230 | conserved hypothetical protein | + | - | - | + |
| BEWA_045020 | conserved hypothetical protein | + | - | - | + |
| BEWA_045290 | conserved hypothetical protein | + | - | - | + |
| BEWA_032100 | cyclophilin 1, putative | + | - | - | + |
| BEWA_043760 | cysteine desulfurase, putative | + | - | - | + |
| BEWA_036120 | cysteinyl-tRNA synthetase, putative | + | - | - | + |
| BEWA_051670 | DEAD box ATP-dependent RNA helicase family member protein | + | - | - | + |
| BEWA_032820 | DEAD box ATP-dependent RNA helicase family member protein | + | - | - | + |
| BEWA_039080 | dephospho-CoA kinase, putative | + | - | - | + |
| BEWA_026820 | DNA gyrase subunit A, putative | + | - | - | + |
| BEWA_043420 | DNA gyrase subunit B, putative | + | - | - | + |
| BEWA_050400 | DNA polymerase family B member protein | + | - | - | + |
| BEWA_028330 | DNA replication licensing factor MCM3, putative | + | - | - | + |
| BEWA_050230 | DNA-directed RNA polymerase, alpha subunit, N terminal domain containing protein | + | - | - | + |
| BEWA_033490 | DNA-directed RNA polymerase, subunit alpha, putative | + | - | - | + |
| BEWA_019850 | DnaJ domain containing protein | + | - | - | + |
| BEWA_029080 | DnaK family member protein | + | - | - | + |
| BEWA_007810 | DNA-repair protein xp-G, putative | + | - | - | + |
| BEWA_042590 | Elongation factor Tu GTP binding domain containing protein | + | - | - | + |
| BEWA_010820 | er lumen protein retaining receptor, putative | + | - | - | + |
| BEWA_009220 | eukaryotic protein of unknown function DUF862 domain containing protein | + | - | - | + |
| BEWA_000920 | ferredoxin, putative | + | - | - | + |
| BEWA_053120 | ferrodoxin reductase-like protein, putative | + | - | - | + |
| BEWA_011100 | glycoprotein endopeptidase, putative | + | - | - | + |
| BEWA_003890 | Got1-like family member protein | + | - | - | + |
| BEWA_001130 | haloacid dehalogenase-like hydrolase family member protein | + | - | - | + |
| BEWA_012170 | haloacid dehalogenase-like hydrolase family member protein | + | - | - | + |
| BEWA_016590 | haloacid dehalogenase-like hydrolase family member protein | + | - | - | + |

| | | | | | |
|---|---|---|---|---|---|
| BEWA_025780 | haloacid dehalogenase-like hydrolase family member protein | + | - | - | + |
| BEWA_035330 | haloacid dehalogenase-like hydrolase family member protein | + | - | - | + |
| BEWA_039640 | haloacid dehalogenase-like hydrolase family member protein | + | - | - | + |
| BEWA_048520 | hexose transporter, putative | + | - | - | + |
| BEWA_021740 | histidyl-tRNA synthetase, putative | + | - | - | + |
| BEWA_046130 | hypothetical protein | + | - | - | + |
| BEWA_046140 | hypothetical protein | + | - | - | + |
| BEWA_047130 | hypothetical protein | + | - | - | + |
| BEWA_047640 | hypothetical protein | + | - | - | + |
| BEWA_047930 | hypothetical protein | + | - | - | + |
| BEWA_048040 | hypothetical protein | + | - | - | + |
| BEWA_048260 | hypothetical protein | + | - | - | + |
| BEWA_048400 | hypothetical protein | + | - | - | + |
| BEWA_049200 | hypothetical protein | + | - | - | + |
| BEWA_049280 | hypothetical protein | + | - | - | + |
| BEWA_049970 | hypothetical protein | + | - | - | + |
| BEWA_050000 | hypothetical protein | + | - | - | + |
| BEWA_050250 | hypothetical protein | + | - | - | + |
| BEWA_051120 | hypothetical protein | + | - | - | + |
| BEWA_051800 | hypothetical protein | + | - | - | + |
| BEWA_052720 | hypothetical protein | + | - | - | + |
| BEWA_053760 | hypothetical protein | + | - | - | + |
| BEWA_054180 | hypothetical protein | + | - | - | + |
| BEWA_054380 | hypothetical protein | + | - | - | + |
| BEWA_054760 | hypothetical protein | + | - | - | + |
| BEWA_054920 | hypothetical protein | + | - | - | + |
| BEWA_000540 | hypothetical protein | + | - | - | + |
| BEWA_000660 | hypothetical protein | + | - | - | + |
| BEWA_000710 | hypothetical protein | + | - | - | + |
| BEWA_001020 | hypothetical protein | + | - | - | + |
| BEWA_001450 | hypothetical protein | + | - | - | + |
| BEWA_001550 | hypothetical protein | + | - | - | + |
| BEWA_002070 | hypothetical protein | + | - | - | + |
| BEWA_002850 | hypothetical protein | + | - | - | + |
| BEWA_003450 | hypothetical protein | + | - | - | + |
| BEWA_003530 | hypothetical protein | + | - | - | + |
| BEWA_004220 | hypothetical protein | + | - | - | + |
| BEWA_004410 | hypothetical protein | + | - | - | + |
| BEWA_007150 | hypothetical protein | + | - | - | + |
| BEWA_007310 | hypothetical protein | + | - | - | + |
| BEWA_009180 | hypothetical protein | + | - | - | + |

| | | | | | |
|---|---|---|---|---|---|
| BEWA_009270 | hypothetical protein | + | - | - | + |
| BEWA_011510 | hypothetical protein | + | - | - | + |
| BEWA_013390 | hypothetical protein | + | - | - | + |
| BEWA_013770 | hypothetical protein | + | - | - | + |
| BEWA_013890 | hypothetical protein | + | - | - | + |
| BEWA_014550 | hypothetical protein | + | - | - | + |
| BEWA_014670 | hypothetical protein | + | - | - | + |
| BEWA_014950 | hypothetical protein | + | - | - | + |
| BEWA_015920 | hypothetical protein | + | - | - | + |
| BEWA_016570 | hypothetical protein | + | - | - | + |
| BEWA_016930 | hypothetical protein | + | - | - | + |
| BEWA_017110 | hypothetical protein | + | - | - | + |
| BEWA_017960 | hypothetical protein | + | - | - | + |
| BEWA_019640 | hypothetical protein | + | - | - | + |
| BEWA_020980 | hypothetical protein | + | - | - | + |
| BEWA_022750 | hypothetical protein | + | - | - | + |
| BEWA_023030 | hypothetical protein | + | - | - | + |
| BEWA_023470 | hypothetical protein | + | - | - | + |
| BEWA_025650 | hypothetical protein | + | - | - | + |
| BEWA_026060 | hypothetical protein | + | - | - | + |
| BEWA_026390 | hypothetical protein | + | - | - | + |
| BEWA_026630 | hypothetical protein | + | - | - | + |
| BEWA_028310 | hypothetical protein | + | - | - | + |
| BEWA_028490 | hypothetical protein | + | - | - | + |
| BEWA_029570 | hypothetical protein | + | - | - | + |
| BEWA_029710 | hypothetical protein | + | - | - | + |
| BEWA_030170 | hypothetical protein | + | - | - | + |
| BEWA_030230 | hypothetical protein | + | - | - | + |
| BEWA_032670 | hypothetical protein | + | - | - | + |
| BEWA_032830 | hypothetical protein | + | - | - | + |
| BEWA_032900 | hypothetical protein | + | - | - | + |
| BEWA_033300 | hypothetical protein | + | - | - | + |
| BEWA_033270 | hypothetical protein | + | - | - | + |
| BEWA_035200 | hypothetical protein | + | - | - | + |
| BEWA_035260 | hypothetical protein | + | - | - | + |
| BEWA_035480 | hypothetical protein | + | - | - | + |
| BEWA_035590 | hypothetical protein | + | - | - | + |
| BEWA_035640 | hypothetical protein | + | - | - | + |
| BEWA_035660 | hypothetical protein | + | - | - | + |
| BEWA_036500 | hypothetical protein | + | - | - | + |
| BEWA_036530 | hypothetical protein | + | - | - | + |

| | | | | | |
|---|---|---|---|---|---|
| BEWA_036950 | hypothetical protein | + | - | - | + |
| BEWA_037130 | hypothetical protein | + | - | - | + |
| BEWA_037270 | hypothetical protein | + | - | - | + |
| BEWA_038740 | hypothetical protein | + | - | - | + |
| BEWA_040750 | hypothetical protein | + | - | - | + |
| BEWA_040820 | hypothetical protein | + | - | - | + |
| BEWA_042660 | hypothetical protein | + | - | - | + |
| BEWA_043110 | hypothetical protein | + | - | - | + |
| BEWA_043330 | hypothetical protein | + | - | - | + |
| BEWA_045600 | hypothetical protein | + | - | - | + |
| BEWA_045950 | hypothetical protein | + | - | - | + |
| BEWA_034150 | kelch domain containing protein | + | - | - | + |
| BEWA_013850 | leucyl-tRNA synthetase, putative | + | - | - | + |
| BEWA_051840 | MAC/Perforin domain containing protein | + | - | - | + |
| BEWA_051910 | MAC/Perforin domain containing protein | + | - | - | + |
| BEWA_018870 | MAC/Perforin domain containing protein | + | - | - | + |
| BEWA_046430 | membrane protein, putative | + | - | - | + |
| BEWA_052620 | membrane protein, putative | + | - | - | + |
| BEWA_000210 | membrane protein, putative | + | - | - | + |
| BEWA_024770 | membrane protein, putative | + | - | - | + |
| BEWA_027080 | membrane protein, putative | + | - | - | + |
| BEWA_031350 | membrane protein, putative | + | - | - | + |
| BEWA_037120 | membrane protein, putative | + | - | - | + |
| BEWA_041210 | membrane protein, putative | + | - | - | + |
| BEWA_000030 | membrane protein, putative | + | - | - | + |
| BEWA_012880 | methionine aminopeptidase, putative | + | - | - | + |
| BEWA_042890 | oxidoreductase NAD-binding domain containing protein | + | - | - | + |
| BEWA_051360 | oxidoreductase, aldo/keto reductase family member protein | + | - | - | + |
| BEWA_030140 | peptide chain release factor 1, putative | + | - | - | + |
| BEWA_022530 | phosphate transporter, putative | + | - | - | + |
| BEWA_000950 | phosphatidate cytidylyltransferase, putative | + | - | - | + |
| BEWA_040010 | phosphatidylinositol 4-kinase family protein | + | - | - | + |
| BEWA_014000 | prohibitin, putative | + | - | - | + |
| BEWA_017540 | prolyl-tRNA synthetase, putative | + | - | - | + |
| BEWA_009910 | protein of unknown function DUF1620 domain containing protein | + | - | - | + |
| BEWA_018620 | pyruvate kinase, putative | + | - | - | + |
| BEWA_037600 | rhoptry-associated protein, putative | + | - | - | + |
| BEWA_050140 | ribosomal protein L21 family member protein | + | - | - | + |
| BEWA_006670 | ribosomal protein S15 domain containing protein | + | - | - | + |
| BEWA_020710 | ribosomal protein S9 family member protein | + | - | - | + |
| BEWA_007260 | RNA modification enzyme, MiaB family domain containing protein | + | - | - | + |

| | | | | | |
|---|---|---|---|---|---|
| BEWA_030580 | serine hydroxymethyltransferase, putative | + | - | - | + |
| BEWA_033130 | seryl-tRNA synthetase, putative | + | - | - | + |
| BEWA_041640 | signal peptidase I family member protein | + | - | - | + |
| BEWA_046070 | signal peptide containing protein | + | - | - | + |
| BEWA_046370 | signal peptide containing protein | + | - | - | + |
| BEWA_046470 | signal peptide containing protein | + | - | - | + |
| BEWA_046490 | signal peptide containing protein | + | - | - | + |
| BEWA_046690 | signal peptide containing protein | + | - | - | + |
| BEWA_046730 | signal peptide containing protein | + | - | - | + |
| BEWA_046860 | signal peptide containing protein | + | - | - | + |
| BEWA_046940 | signal peptide containing protein | + | - | - | + |
| BEWA_047000 | signal peptide containing protein | + | - | - | + |
| BEWA_047090 | signal peptide containing protein | + | - | - | + |
| BEWA_047340 | signal peptide containing protein | + | - | - | + |
| BEWA_047400 | signal peptide containing protein | + | - | - | + |
| BEWA_047530 | signal peptide containing protein | + | - | - | + |
| BEWA_047790 | signal peptide containing protein | + | - | - | + |
| BEWA_047810 | signal peptide containing protein | + | - | - | + |
| BEWA_047830 | signal peptide containing protein | + | - | - | + |
| BEWA_047950 | signal peptide containing protein | + | - | - | + |
| BEWA_048420 | signal peptide containing protein | + | - | - | + |
| BEWA_048540 | signal peptide containing protein | + | - | - | + |
| BEWA_048620 | signal peptide containing protein | + | - | - | + |
| BEWA_048800 | signal peptide containing protein | + | - | - | + |
| BEWA_048840 | signal peptide containing protein | + | - | - | + |
| BEWA_049130 | signal peptide containing protein | + | - | - | + |
| BEWA_049310 | signal peptide containing protein | + | - | - | + |
| BEWA_049470 | signal peptide containing protein | + | - | - | + |
| BEWA_049550 | signal peptide containing protein | + | - | - | + |
| BEWA_049640 | signal peptide containing protein | + | - | - | + |
| BEWA_049890 | signal peptide containing protein | + | - | - | + |
| BEWA_050300 | signal peptide containing protein | + | - | - | + |
| BEWA_050550 | signal peptide containing protein | + | - | - | + |
| BEWA_050700 | signal peptide containing protein | + | - | - | + |
| BEWA_051340 | signal peptide containing protein | + | - | - | + |
| BEWA_051890 | signal peptide containing protein | + | - | - | + |
| BEWA_052230 | signal peptide containing protein | + | - | - | + |
| BEWA_052330 | signal peptide containing protein | + | - | - | + |
| BEWA_053680 | signal peptide containing protein | + | - | - | + |
| BEWA_053880 | signal peptide containing protein | + | - | - | + |
| BEWA_053930 | signal peptide containing protein | + | - | - | + |

| | | | | | |
|---|---|---|---|---|---|
| BEWA_053940 | signal peptide containing protein | + | - | - | + |
| BEWA_053980 | signal peptide containing protein | + | - | - | + |
| BEWA_054220 | signal peptide containing protein | + | - | - | + |
| BEWA_054590 | signal peptide containing protein | + | - | - | + |
| BEWA_000280 | signal peptide containing protein | + | - | - | + |
| BEWA_000340 | signal peptide containing protein | + | - | - | + |
| BEWA_000500 | signal peptide containing protein | + | - | - | + |
| BEWA_000510 | signal peptide containing protein | + | - | - | + |
| BEWA_000670 | signal peptide containing protein | + | - | - | + |
| BEWA_000800 | signal peptide containing protein | + | - | - | + |
| BEWA_000810 | signal peptide containing protein | + | - | - | + |
| BEWA_000850 | signal peptide containing protein | + | - | - | + |
| BEWA_000900 | signal peptide containing protein | + | - | - | + |
| BEWA_000910 | signal peptide containing protein | + | - | - | + |
| BEWA_001010 | signal peptide containing protein | + | - | - | + |
| BEWA_001670 | signal peptide containing protein | + | - | - | + |
| BEWA_001890 | signal peptide containing protein | + | - | - | + |
| BEWA_001950 | signal peptide containing protein | + | - | - | + |
| BEWA_002120 | signal peptide containing protein | + | - | - | + |
| BEWA_002390 | signal peptide containing protein | + | - | - | + |
| BEWA_002570 | signal peptide containing protein | + | - | - | + |
| BEWA_002770 | signal peptide containing protein | + | - | - | + |
| BEWA_002940 | signal peptide containing protein | + | - | - | + |
| BEWA_003120 | signal peptide containing protein | + | - | - | + |
| BEWA_003440 | signal peptide containing protein | + | - | - | + |
| BEWA_003540 | signal peptide containing protein | + | - | - | + |
| BEWA_003650 | signal peptide containing protein | + | - | - | + |
| BEWA_003760 | signal peptide containing protein | + | - | - | + |
| BEWA_003830 | signal peptide containing protein | + | - | - | + |
| BEWA_003900 | signal peptide containing protein | + | - | - | + |
| BEWA_004140 | signal peptide containing protein | + | - | - | + |
| BEWA_004210 | signal peptide containing protein | + | - | - | + |
| BEWA_004480 | signal peptide containing protein | + | - | - | + |
| BEWA_005720 | signal peptide containing protein | + | - | - | + |
| BEWA_006750 | signal peptide containing protein | + | - | - | + |
| BEWA_007290 | signal peptide containing protein | + | - | - | + |
| BEWA_007300 | signal peptide containing protein | + | - | - | + |
| BEWA_008870 | signal peptide containing protein | + | - | - | + |
| BEWA_009040 | signal peptide containing protein | + | - | - | + |
| BEWA_012250 | signal peptide containing protein | + | - | - | + |
| BEWA_012590 | signal peptide containing protein | + | - | - | + |

| | | | | | |
|---|---|---|---|---|---|
| BEWA_012810 | signal peptide containing protein | + | - | - | + |
| BEWA_012860 | signal peptide containing protein | + | - | - | + |
| BEWA_012900 | signal peptide containing protein | + | - | - | + |
| BEWA_013050 | signal peptide containing protein | + | - | - | + |
| BEWA_013410 | signal peptide containing protein | + | - | - | + |
| BEWA_013460 | signal peptide containing protein | + | - | - | + |
| BEWA_013910 | signal peptide containing protein | + | - | - | + |
| BEWA_015040 | signal peptide containing protein | + | - | - | + |
| BEWA_015770 | signal peptide containing protein | + | - | - | + |
| BEWA_015820 | signal peptide containing protein | + | - | - | + |
| BEWA_015860 | signal peptide containing protein | + | - | - | + |
| BEWA_016310 | signal peptide containing protein | + | - | - | + |
| BEWA_016480 | signal peptide containing protein | + | - | - | + |
| BEWA_017060 | signal peptide containing protein | + | - | - | + |
| BEWA_017170 | signal peptide containing protein | + | - | - | + |
| BEWA_017180 | signal peptide containing protein | + | - | - | + |
| BEWA_017330 | signal peptide containing protein | + | - | - | + |
| BEWA_019310 | signal peptide containing protein | + | - | - | + |
| BEWA_020850 | signal peptide containing protein | + | - | - | + |
| BEWA_022100 | signal peptide containing protein | + | - | - | + |
| BEWA_022160 | signal peptide containing protein | + | - | - | + |
| BEWA_023080 | signal peptide containing protein | + | - | - | + |
| BEWA_023600 | signal peptide containing protein | + | - | - | + |
| BEWA_023750 | signal peptide containing protein | + | - | - | + |
| BEWA_024350 | signal peptide containing protein | + | - | - | + |
| BEWA_025140 | signal peptide containing protein | + | - | - | + |
| BEWA_025340 | signal peptide containing protein | + | - | - | + |
| BEWA_025540 | signal peptide containing protein | + | - | - | + |
| BEWA_025600 | signal peptide containing protein | + | - | - | + |
| BEWA_025700 | signal peptide containing protein | + | - | - | + |
| BEWA_025770 | signal peptide containing protein | + | - | - | + |
| BEWA_025840 | signal peptide containing protein | + | - | - | + |
| BEWA_025900 | signal peptide containing protein | + | - | - | + |
| BEWA_026410 | signal peptide containing protein | + | - | - | + |
| BEWA_027420 | signal peptide containing protein | + | - | - | + |
| BEWA_027440 | signal peptide containing protein | + | - | - | + |
| BEWA_027480 | signal peptide containing protein | + | - | - | + |
| BEWA_027540 | signal peptide containing protein | + | - | - | + |
| BEWA_027900 | signal peptide containing protein | + | - | - | + |
| BEWA_028050 | signal peptide containing protein | + | - | - | + |
| BEWA_028140 | signal peptide containing protein | + | - | - | + |

| | | | | | |
|---|---|---|---|---|---|
| BEWA_028220 | signal peptide containing protein | + | - | - | + |
| BEWA_028250 | signal peptide containing protein | + | - | - | + |
| BEWA_028260 | signal peptide containing protein | + | - | - | + |
| BEWA_028630 | signal peptide containing protein | + | - | - | + |
| BEWA_028780 | signal peptide containing protein | + | - | - | + |
| BEWA_029050 | signal peptide containing protein | + | - | - | + |
| BEWA_029330 | signal peptide containing protein | + | - | - | + |
| BEWA_029340 | signal peptide containing protein | + | - | - | + |
| BEWA_029380 | signal peptide containing protein | + | - | - | + |
| BEWA_029430 | signal peptide containing protein | + | - | - | + |
| BEWA_029460 | signal peptide containing protein | + | - | - | + |
| BEWA_029510 | signal peptide containing protein | + | - | - | + |
| BEWA_029630 | signal peptide containing protein | + | - | - | + |
| BEWA_029960 | signal peptide containing protein | + | - | - | + |
| BEWA_030020 | signal peptide containing protein | + | - | - | + |
| BEWA_030190 | signal peptide containing protein | + | - | - | + |
| BEWA_031110 | signal peptide containing protein | + | - | - | + |
| BEWA_031310 | signal peptide containing protein | + | - | - | + |
| BEWA_031700 | signal peptide containing protein | + | - | - | + |
| BEWA_031770 | signal peptide containing protein | + | - | - | + |
| BEWA_032220 | signal peptide containing protein | + | - | - | + |
| BEWA_032290 | signal peptide containing protein | + | - | - | + |
| BEWA_034030 | signal peptide containing protein | + | - | - | + |
| BEWA_034140 | signal peptide containing protein | + | - | - | + |
| BEWA_034520 | signal peptide containing protein | + | - | - | + |
| BEWA_035300 | signal peptide containing protein | + | - | - | + |
| BEWA_035650 | signal peptide containing protein | + | - | - | + |
| BEWA_035670 | signal peptide containing protein | + | - | - | + |
| BEWA_035710 | signal peptide containing protein | + | - | - | + |
| BEWA_035840 | signal peptide containing protein | + | - | - | + |
| BEWA_035860 | signal peptide containing protein | + | - | - | + |
| BEWA_035870 | signal peptide containing protein | + | - | - | + |
| BEWA_035970 | signal peptide containing protein | + | - | - | + |
| BEWA_036210 | signal peptide containing protein | + | - | - | + |
| BEWA_036380 | signal peptide containing protein | + | - | - | + |
| BEWA_036520 | signal peptide containing protein | + | - | - | + |
| BEWA_036840 | signal peptide containing protein | + | - | - | + |
| BEWA_037460 | signal peptide containing protein | + | - | - | + |
| BEWA_037470 | signal peptide containing protein | + | - | - | + |
| BEWA_038380 | signal peptide containing protein | + | - | - | + |
| BEWA_038570 | signal peptide containing protein | + | - | - | + |

| | | | | | |
|---|---|---|---|---|---|
| BEWA_039180 | signal peptide containing protein | + | - | - | + |
| BEWA_039270 | signal peptide containing protein | + | - | - | + |
| BEWA_039440 | signal peptide containing protein | + | - | - | + |
| BEWA_039790 | signal peptide containing protein | + | - | - | + |
| BEWA_040050 | signal peptide containing protein | + | - | - | + |
| BEWA_040090 | signal peptide containing protein | + | - | - | + |
| BEWA_041360 | signal peptide containing protein | + | - | - | + |
| BEWA_041380 | signal peptide containing protein | + | - | - | + |
| BEWA_041710 | signal peptide containing protein | + | - | - | + |
| BEWA_041870 | signal peptide containing protein | + | - | - | + |
| BEWA_044370 | signal peptide containing protein | + | - | - | + |
| BEWA_044530 | signal peptide containing protein | + | - | - | + |
| BEWA_000040 | signal peptide containing protein | + | - | - | + |
| BEWA_045420 | signal peptide containing protein | + | - | - | + |
| BEWA_045700 | signal peptide containing protein | + | - | - | + |
| BEWA_020540 | tRNA 5-methylaminomethyl-2-thiouridylate-methyltransferase, putative | + | - | - | + |
| BEWA_005420 | tRNA-pseudouridine synthase I, putative | + | - | - | + |
| BEWA_038330 | tyrosyl-tRNA synthetase, putative | + | - | - | + |
| BEWA_054010 | zinc finger protein DHHC domain containing protein | + | - | - | + |
| BEWA_026980 | zinc finger protein DHHC domain containing protein | + | - | - | + |

[a] Has a signal peptide as predicted by SignalP, Philius or Phobius.
[b] Belongs to a pathway predicted to be in the apicoplast
[c] Contains an apicoplast targeting domain as predicted by PlasmoAP
[d] Contains an apicoplast targeting domain as predicted by ApicoAP
