# Supplementary material for: Comparative genomic analysis and phylogenetic position of Theileria equi
Source: BMC Genomics. 2012 Nov 9;13:603. doi: 10.1186/1471-2164-13-603 (PMC3505731; doi:10.1186/1471-2164-13-603)
Supplement: Additional file 3 — Table. GPI anchored proteins predicted by GPI-SOM. [file 1471-2164-13-603-S3.pdf]

Table: GPI anchored proteins predicted by GPI-SOM

Gene Identifier	Potential Cleavage Site
BEWA_000030	--
BEWA_000340	Best--match--for--cleavage--site--at--C-13]
BEWA_000850	--
BEWA_001010	--
BEWA_001230	Best--match--for--cleavage--site--at--C-22]
BEWA_002200	Best--match--for--cleavage--site--at--C-22]
BEWA_002380	Best--match--for--cleavage--site--at--C-22]
BEWA_002390	--
BEWA_002580	Best--match--for--cleavage--site--at--C-5]
BEWA_003390	--
BEWA_003540	Best--match--for--cleavage--site--at--C-24]
BEWA_003650	--
BEWA_003870	Best--match--for--cleavage--site--at--C-22]
BEWA_004370	--
BEWA_004410	Best--match--for--cleavage--site--at--C-25]
BEWA_005180	--
BEWA_005720	Best--match--for--cleavage--site--at--C-5]
BEWA_007020	Best--match--for--cleavage--site--at--C-28]
BEWA_008040	--
BEWA_008790	Best--match--for--cleavage--site--at--C-29]
BEWA_008910	Best--match--for--cleavage--site--at--C-22]
BEWA_012600	Best--match--for--cleavage--site--at--C-23]
BEWA_012810	Best--match--for--cleavage--site--at--C-7]
BEWA_013910	Best--match--for--cleavage--site--at--C-13]
BEWA_014340	Best--match--for--cleavage--site--at--C-30]
BEWA_016570	Best--match--for--cleavage--site--at--C-17]
BEWA_016670	Best--match--for--cleavage--site--at--C-16]
BEWA_016830	Best--match--for--cleavage--site--at--C-21]
BEWA_016880	Best--match--for--cleavage--site--at--C-19]
BEWA_017190	--
BEWA_017320	Best--match--for--cleavage--site--at--C-13]
BEWA_017540	Best--match--for--cleavage--site--at--C-16]
BEWA_017860	Best--match--for--cleavage--site--at--C-32]
BEWA_019720	--
BEWA_020830	Best--match--for--cleavage--site--at--C-3]
BEWA_021550	--
BEWA_022160	Best--match--for--cleavage--site--at--C-31]
BEWA_022380	Best--match--for--cleavage--site--at--C-15]
BEWA_022640	--
BEWA_022740	--
BEWA_023080	--
BEWA_023390	Best--match--for--cleavage--site--at--C-23]
BEWA_023400	--

BEWA_023410	--
BEWA_023510	Best--match--for--cleavage--site--at--C-22]
BEWA_024410	--
BEWA_024470	--
BEWA_024600	Best--match--for--cleavage--site--at--C-19]
BEWA_025340	--
BEWA_025600	Best--match--for--cleavage--site--at--C-17]
BEWA_025890	Best--match--for--cleavage--site--at--C-9]
BEWA_026380	Best--match--for--cleavage--site--at--C-25]
BEWA_026550	--
BEWA_026570	--
BEWA_026850	--
BEWA_027080	--
BEWA_027140	Best--match--for--cleavage--site--at--C-32]
BEWA_027270	Best--match--for--cleavage--site--at--C-30]
BEWA_027540	Best--match--for--cleavage--site--at--C-16]
BEWA_027630	Best--match--for--cleavage--site--at--C-22]
BEWA_027750	--
BEWA_028210	--
BEWA_028490	Best--match--for--cleavage--site--at--C-18]
BEWA_028640	Best--match--for--cleavage--site--at--C-32]
BEWA_028720	Best--match--for--cleavage--site--at--C-27]
BEWA_030350	--
BEWA_031280	--
BEWA_031520	--
BEWA_031730	Best--match--for--cleavage--site--at--C-29]
BEWA_031750	Best--match--for--cleavage--site--at--C-13]
BEWA_031810	Best--match--for--cleavage--site--at--C-32]
BEWA_032950	--
BEWA_033100	Best--match--for--cleavage--site--at--C-4]
BEWA_033780	Best--match--for--cleavage--site--at--C-32]
BEWA_033790	Best--match--for--cleavage--site--at--C-25]
BEWA_034150	Best--match--for--cleavage--site--at--C-23]
BEWA_034340	Best--match--for--cleavage--site--at--C-13]
BEWA_034840	--
BEWA_035120	--
BEWA_035180	Best--match--for--cleavage--site--at--C-20]
BEWA_035240	--
BEWA_035360	Best--match--for--cleavage--site--at--C-25]
BEWA_035420	Best--match--for--cleavage--site--at--C-24]
BEWA_036210	Best--match--for--cleavage--site--at--C-19]
BEWA_036380	Best--match--for--cleavage--site--at--C-30]
BEWA_037260	--
BEWA_037460	Best--match--for--cleavage--site--at--C-27]
BEWA_037820	Best--match--for--cleavage--site--at--C-21]

BEWA_038380	Best--match--for--cleavage--site--at--C-10]
BEWA_038950	Best--match--for--cleavage--site--at--C-24]
BEWA_039640	Best--match--for--cleavage--site--at--C-16]
BEWA_039900	--
BEWA_040440	Best--match--for--cleavage--site--at--C-10]
BEWA_040850	--
BEWA_041210	--
BEWA_041660	--
BEWA_042700	Best--match--for--cleavage--site--at--C-4]
BEWA_043230	--
BEWA_044000	Best--match--for--cleavage--site--at--C-17]
BEWA_044360	Best--match--for--cleavage--site--at--C-29]
BEWA_044910	Best--match--for--cleavage--site--at--C-17]
BEWA_045090	--
BEWA_045350	--
BEWA_045790	Best--match--for--cleavage--site--at--C-23]
BEWA_046160	--
BEWA_046430	Best--match--for--cleavage--site--at--C-23]
BEWA_046760	--
BEWA_047010	--
BEWA_047280	--
BEWA_047350	Best--match--for--cleavage--site--at--C-19]
BEWA_047690	--
BEWA_047960	Best--match--for--cleavage--site--at--C-24]
BEWA_048540	Best--match--for--cleavage--site--at--C-32]
BEWA_048750	Best--match--for--cleavage--site--at--C-4]
BEWA_048790	--
BEWA_049310	--
BEWA_049960	Best--match--for--cleavage--site--at--C-29]
BEWA_049970	--
BEWA_050300	Best--match--for--cleavage--site--at--C-23]
BEWA_051110	Best--match--for--cleavage--site--at--C-25]
BEWA_051920	--
BEWA_052050	Best--match--for--cleavage--site--at--C-26]
BEWA_052240	--
BEWA_052710	--
BEWA_052730	--
BEWA_053490	Best--match--for--cleavage--site--at--C-25]
BEWA_053530	--
BEWA_053600	--
BEWA_053920	Best--match--for--cleavage--site--at--C-25]
BEWA_054200	--
BEWA_054380	Best--match--for--cleavage--site--at--C-22]
BEWA_054930	Best--match--for--cleavage--site--at--C-25]
