# Supplementary material for: Comparative genomic analysis and phylogenetic position of Theileria equi
Source: BMC Genomics. 2012 Nov 9;13:603. doi: 10.1186/1471-2164-13-603 (PMC3505731; doi:10.1186/1471-2164-13-603)
Supplement: Additional file 4 — Table. Transporter comparison B.bovis, T. equi, and T.parva. [file 1471-2164-13-603-S4.pdf]

Table: Transporter comparison *B. bovis*, *T. equi*, and *T. parva*

	***B. bovis* T2Bo**	***T. equi***	***T. parva***
**Genome Size(Mb):**	8.2	11.7	8.4
**Total Transporter Proteins:**	78	142	85
**No. of Transporters per Mb genome:**	**9.51**	**12.14**	**10.12**
**ATP-Dependent**	**33 (42%)**	**81 (57%)**	**40 (47%)**
The ATP-binding Cassette (ABC) Superfamily	9	45	17
The Arsenite-Antimonite (ArsAB) Efflux Family	1	1	1
The Chloroplast Envelope Protein Translocase (CEPT or Tic-Toc) Family	0	2	0
The H+- or Na+-translocating F-type, V-type and A-type ATPase (F-ATPase) Superfamily	2	2	2
The H+-translocating Pyrophosphatase (H+-PPase) Family	0	0	1
The Type II (General) Secretory Pathway (IISP) Family	3	11	3
The Mitochondrial Protein Translocase (MPT) Family	10	12	9
The P-type ATPase (P-ATPase) Superfamily	8	8	7
The Septal DNA Translocator (S-DNA-T) Family	0	0	0
**Ion Channels**	**7 (9%)**	**8 (6%)**	**5 (6%)**
The ATP-gated Cation Channel (ACC) Family	0	0	0
The Ammonia Transporter Channel (Amt) Family	0	0	0
The Annexin (Annexin) Family	0	0	0
The Bcl-2 (Bcl-2) Family	0	0	0
The Anion Channel-forming Bestrophin (Bestrophin) Family	0	0	0
The BCL2/Adenovirus E1B-interacting Protein 3 (BNip3) Family	0	0	0
The CD20 Ca2+ Channel (CD20) Family	0	0	0
The Intracellular Chloride Channel (CLIC) Family	0	0	0
The Gap Junction-forming Connexin (Connexin) Family	0	0	0
The Ca2+ Release-activated Ca2+ (CRAC) Channel (CRAC-C) Family	0	0	0
The Copper Transporter (Ctr) Family	1	1	1
The Epithelial Chloride Channel (E-ClC) Family	0	0	0
The Epithelial Na+ Channel (ENaC) Family	0	0	0
The Glutamate-gated Ion Channel (GIC) Family of Neurotransmitter Receptors	0	0	0
The Intracellular Chloride Channel (ICC) Family	0	0	0
The Gap Junction-forming Innexin (Innexin) Family	0	0	0
The Inward Rectifier K+ Channel (IRK-C) Family	0	0	0
The Neurotransmitter Receptor, Cys loop, Ligand-gated Ion Channel (LIC) Family	0	0	0
The Yeast Stretch-Activated, Cation-Selective, Ca2+ Channel, Mid1 (Mid1) Family	0	0	0
The Major Intrinsic Protein (MIP) Family	0	0	0
The CorA Metal Ion Transporter (MIT) Family	0	1	0
The Large Conductance Mechanosensitive Ion Channel (MscL) Family	0	0	0

The Small Conductance Mechanosensitive Ion Channel (MscS) Family	2	2	2
The Non-selective Cation Channel-2 (NSCC2) Family	1	1	1
The Polycystin Cation Channel (PCC) Family	0	0	0
The Plasmolipin (Plasmolipin) Family	0	0	0
The Phospholamban (Ca2+-channel and Ca2+-ATPase Regulator) (PLB) Family	0	0	0
The Phospholemman (PLM) Family	0	0	0
The Presenilin ER Ca2+ Leak Channel (Presenilin) Family	0	0	0
The Ryanodine-Inositol 1,4,5-triphosphate Receptor Ca2+ Channel (RIR-CaC) Family	0	0	0
The Chloroplast Envelope Anion Channel-forming Tic110 (Tic110) Family	0	0	0
The Homotrimeric Cation Channel (TRIC) Family	0	0	0
The Transient Receptor Potential Ca2+ Channel (TRP-CC) Family	0	0	0
The Anion Channel Tweety (Tweety) Family	0	0	0
The Urea/Amide Channel (UAC) Family	0	0	0
The Urea Transporter (UT) Family	0	0	0
The Voltage-gated Ion Channel (VIC) Superfamily	3	3	1
**Phosphotransferase System (PTS)**	**0 (0%)**	**0 (0%)**	**0 (0%)**
General PTS	0	0	0
Sugar Specific PTS	0	0	0
**Secondary Transporter**	**38 (49%)**	**51 (36%)**	**40 (47%)**
The 2-Hydroxycarboxylate Transporter (2-HCT) Family	0	0	0
The ATP:ADP Antiporter (AAA) Family	0	0	0
The Amino Acid/Auxin Permease (AAAP) Family	0	0	0
The Aspartate:Alanine Exchanger (AAE) Family	0	0	0
The p-Aminobenzoyl-glutamate Transporter (AbgT) Family	0	0	0
The Arsenical Resistance-3 (ACR3) Family	0	0	0
The Anion Exchanger (AE) Family	0	0	0
The Auxin Efflux Carrier (AEC) Family	0	0	0
The Alanine or Glycine:Cation Symporter (AGCS) Family	0	0	0
The Autoinducer-2 Exporter (AI-2E) Family (Formerly the PerM Family, TC #9.B.22)	0	0	0
The Amino Acid-Polyamine-Organocation (APC) Family	0	0	0
The Aromatic Acid Exporter (ArAE) Family	0	0	0
The Arsenite-Antimonite (ArsB) Efflux Family	0	0	0
The Short Chain Fatty Acid Uptake (AtoE) Family	0	0	0
The Bile Acid:Na+ Symporter (BASS) Family	0	0	0
The Betaine/Carnitine/Choline Transporter (BCCT) Family	0	0	0
The Benzoate:H+ Symporter (BenE) Family	0	0	0
The Biotin Uptake Transporter (BUT) Family	0	0	0
The Ca2+:Cation Antiporter (CaCA) Family	0	0	0
The Cadmium Resistance (CadD) Family	0	0	0

The Cation-Chloride Cotransporter (CCC) Family	0	0	0
The Cation Diffusion Facilitator (CDF) Family	1	0	0
The Chromate Ion Transporter (CHR) Family	0	0	0
The Citrate-Mg2+:H+ (CitM) Citrate-Ca2+:H+ (CitH) Symporter (CitMHS) Family	0	0	0
The Chloride Carrier/Channel (ClC) Family	0	0	0
The Concentrative Nucleoside Transporter (CNT) Family	0	0	0
The Monovalent Cation:Proton Antiporter-1 (CPA1) Family	2	1	1
The Monovalent Cation:Proton Antiporter-2 (CPA2) Family	0	0	0
The Monovalent Cation (K+ or Na+):Proton Antiporter-3 (CPA3) Family	0	0	0
The Choline Transporter Like (CTL) Family	0	0	0
The Dicarboxylate/Amino Acid:Cation (Na+ or H+) Symporter (DAACS) Family	0	1	1
The Divalent Anion:Na+ Symporter (DASS) Family	0	0	0
The C4-Dicarboxylate Uptake (Dcu) Family	0	0	0
The C4-dicarboxylate Uptake C (DcuC) Family	0	0	0
The Drug/Metabolite Transporter (DMT) Superfamily	7	4	4
The Equilibrative Nucleoside Transporter (ENT) Family	0	0	0
The Glutamate:Na+ Symporter (ESS) Family	0	0	0
The Folate-Biopterin Transporter (FBT) Family	1	5	2
The Formate-Nitrite Transporter (FNT) Family	1	1	1
The Gluconate:H+ Symporter (GntP) Family	0	0	0
The Glycoside-Pentoside-Hexuronide (GPH):Cation Symporter Family	0	0	0
The Glycerol Uptake (GUP) Family	0	0	0
The Hydroxy/Aromatic Amino Acid Permease (HAAAP) Family	0	0	0
The 2-Keto-3-Deoxygluconate Transporter (KDGT) Family	0	0	0
The K+ Uptake Permease (KUP) Family	0	0	0
The Lysosomal Cystine Transporter (LCT) Family	0	0	0
The Lactate Permease (LctP) Family	0	0	0
The Branched Chain Amino Acid Exporter (LIV-E) Family	0	0	0
The Branched Chain Amino Acid:Cation Symporter (LIVCS) Family	0	0	0
The L-Lysine Exporter (LysE) Family	0	0	0
The Mitochondrial Carrier (MC) Family	7	9	9
The 4 TMS Multidrug Endosomal Transporter (MET) Family	0	0	0
The Chloroplast Maltose Exporter (MEX) Family	0	0	0
The Major Facilitator Superfamily (MFS)	13	23	16
The Multidrug/Oligosaccharidyl-lipid/Polysaccharide (MOP) Flippase Superfamily	0	0	0
The Malonate:Na+ Symporter (MSS) Family	0	0	0
The Mitochondrial Tricarboxylate Carrier (MTC) Family	0	0	0

The Nucleobase:Cation Symporter-1 (NCS1) Family	0	0	0
The Nucleobase:Cation Symporter-2 (NCS2) Family	0	0	0
The NhaA Na+:H+ Antiporter (NhaA) Family	0	0	0
The NhaB Na+:H+ Antiporter (NhaB) Family	0	0	0
The NhaC Na+:H+ Antiporter (NhaC) Family	0	0	0
The NhaD Na+:H+ Antiporter (NhaD) Family	0	0	0
The Ni2+-Co2+ Transporter (NiCoT) Family	0	0	0
The Metal Ion (Mn2+-iron) Transporter (Nramp) Family	0	0	0
The Neurotransmitter:Sodium Symporter (NSS) Family	1	1	1
The Organo Anion Transporter (OAT) Family	0	0	0
The Oligopeptide Transporter (OPT) Family	0	0	0
The Organic Solute Transporter (OST) Family	0	0	0
The Cytochrome Oxidase Biogenesis (Oxa1) Family	1	1	1
The Inorganic Phosphate Transporter (PiT) Family	1	1	1
The Phosphate:Na+ Symporter (PNaS) Family	0	0	0
The Proton-dependent Oligopeptide Transporter (POT) Family	0	0	0
The Reduced Folate Carrier (RFC) Family	0	0	0
The Riboflavin Transporter (RFT) Family	0	0	0
The Resistance to Homoserine/Threonine (RhtB) Family	0	0	0
The Resistance-Nodulation-Cell Division (RND) Superfamily	0	0	0
The Na+-dependent Bicarbonate Transporter (SBT) Family	0	0	0
The Silicon Transporter (Sit) Family	0	0	0
The Solute:Sodium Symporter (SSS) Family	0	0	0
The Vitamin A Receptor/Transporter (STRA6) Family	0	0	0
The Sulfate Permease (SulP) Family	1	1	1
The Twin Arginine Targeting (Tat) Family	0	0	0
The Telurite-resistance/Dicarboxylate Transporter (TDT) Family	0	0	0
The Threonine/Serine Exporter (ThrE) Family	0	0	0
The Tripartite ATP-independent Periplasmic Transporter (TRAP-T) Family	0	0	0
The K+ Transporter (Trk) Family	0	0	0
The Tricarboxylate Transporter (TTT) Family	0	0	0
The Vacuolar Iron Transporter (VIT) Family	0	1	0
The Zinc (Zn2+)-Iron (Fe2+) Permease (ZIP) Family	2	2	2
**Unclassified**	**0 (0%)**	**2 (1%)**	**0 (0%)**
The ATP Exporter (ATP-E) Family	0	0	0
The Capsular Polysaccharide Exporter (CPS-E) Family	0	0	0
The Double Stranded RNA Transporter (dsRNA-T) Family	0	0	0

The Ethanolamine Facilitator (EAF) Family	0	0	0
The Ferrous Iron Uptake (FeoB) Family	0	0	0
The Low Affinity Fe2+ Transporter (FeT) Family	0	0	0
The Ferroportin (FP) Family	0	0	0
The Peptidoglycolipid Addressing Protein (GAP) Family	0	0	0
The HlyC/CorC (HCC) Family	0	1	0
The Iron/Lead Transporter (ILT) Superfamily	0	0	0
The Lysosomal Protein Import (LPI) Family	0	0	0
The Lipid-translocating Exporter (LTE) Family	0	0	0
The Magnesium Transporter1 (MagT1) Family	0	0	0
The MerTP Mercuric Ion (Hg2+) Permease (MerTP) Family	0	0	0
The Mg2+ Transporter-E (MgtE) Family	0	0	0
The Non ABC Multidrug Exporter (N-MDE) Family	0	0	0
The NIPA Mg2+ Uptake Permease (NIPA) Family	0	0	0
The Peroxisomal Protein Importer (PPI) Family	0	0	0
The Integral Membrane Peroxisomal Protein Importer-2 (PPI2) Family	0	0	0
The Putative Peptide Uptake or Activated Fatty Acid Export Permease (PUP) Family	0	0	0
The SdpC (Peptide-Antibiotic Killer Factor) Immunity Protein, SdpI (SdpI) Family	0	0	0
The Tellurium Ion Resistance (TerC) Family	0	0	0
The Putative 4-Toluene Sulfonate Uptake Permease (TSUP) Family	0	0	0
The YggT or Fanciful K+ Uptake-B (FkuB; YggT) Family	0	1	0
