# Supplementary material for: Comparative genomic analysis and phylogenetic position of Theileria equi
Source: BMC Genomics. 2012 Nov 9;13:603. doi: 10.1186/1471-2164-13-603 (PMC3505731; doi:10.1186/1471-2164-13-603)
Supplement: Additional file 5 — Table. 30 largest protein families. [file 1471-2164-13-603-S5.pdf]

Table: 30 largest protein families

Family ID	Number Included	Notable Properties: Domains, Homologs or Characteristics
1	356	42% of Members Contain FAINT Domain (PF04385)
2	319	90% of Members Annotated as Hypothetical Proteins
3	109	91% of Members Share Sequence Identity to Tar-like sequences
4	92	95% of Members Annotated as Hypothetical Proteins
5	91	100% of Members Annotated as Hypothetical Proteins
6	90	55% of Members Contain FAINT Domain (PF04385), 33% of Members Have Signal Peptide
7	59	49% of Members Contain FAINT Domain (PF04385)
8	56	64% of members Have Signal Peptide
9	49	100% of Members Annotated as Hypothetical Proteins
10	42	95% of Members Annotated as Hypothetical Proteins
11	42	81% of Members Contain ABC Transporter Domain (PF00005)
12	37	81% of Members Contain Protein Kinase Domain (PF00069)
13	31	100% of Members Contain Helicase Conserved C-Terminal Domain (PF00271), 94% Contain DEAD/DEAH Box Helicase Domain (PF00270)
14	28	71% of Members Have Signal Peptide
15	28	100% of Members Annotated as Hypothetical Proteins
16	26	100% of Members Contain Haloacid Dehalogenase-Like Hydrolase Domain (PF08282)
17	24	38% of Members Contain Reverse Transcriptase (RNA-Dependent DNA Polymerase) Domain (PF00078)
18	24	100% Share Marginal Sequence Identity to Hypothetical Protein in Theileria annulata
19	24	96% of Members Annotated as Hypothetical Proteins
20	20	60% of Members Have Signal Peptide
21	17	100% of Members Contain ATPase, AAA Family Domain (PF00004)
22	14	86% of Members Annotated as Hypothetical Proteins
23	14	100% of Members Have Signal Peptide
24	12	58% of Members Have Signal Peptide, 50% of Members Contain FAINT Domain (PF04385)
25	12	92% of Members Contain Peptidyl-Prolyl cis-trans Isomerase Cyclophilin-Type Domain (PF00160)
26	11	91% of Members Contain Protein Kinase Domain (PF00069)
27	11	91% of Members Contain DnaJ Domain (PF00226)
28	11	100% of Members Contain Ubiquitin-Conjugating Enzyme Domain (PF00179)
29	10	EMA Family, 80% of Members Contain Merozoite Antigen Domain (PF02488)
30	10	100% of Members Contain Ras Family Domain (PF00071) and Miro-Like Protein Domain (PF08477)
