# Supplementary material for: Comparative genomic analysis and phylogenetic position of Theileria equi
Source: BMC Genomics. 2012 Nov 9;13:603. doi: 10.1186/1471-2164-13-603 (PMC3505731; doi:10.1186/1471-2164-13-603)
Supplement: Additional file 6 — Figure. Alignment of EMA family sequences. Residues highlighted in yellow are conserved among all family members, and those in blue conserved among the majority of family members. Dashes represent gaps introduced to accommodate non-conserved stretches of sequence. [file 1471-2164-13-603-S6.pdf]

```
                              1                                                                                                            110
BEWA_047350 EMA10   (1) MMTKSLFGVAVFTYFAVSSVFAAAQPSPQELKPVAGPDGLQGPIGPVGKGLTAKTIAKPDPGSARELSSGANSTHDRESTGKPEESERRYDGPGSAGFCPGGPTNPSAFI
BEWA_026850 EMA1    (1) MI--SKSFAFVFASIAISSILAEEEKPKASG--------------------AVVDFQLESIDHVTIDKQSE-E---HIVYTAHEGYAVEKVKEGDSVIKTFDLKEQTPKT
BEWA_017320 EMA2    (1) MLSKSFAGVFTLAYLAVSGIFADEAPKVSG---------------------AVVTLGATTLDHITVDDTTAG----KVVYTAHSGYAVEKVVDGDKVVKAFDLKVDAPRS
BEWA_034340 EMA3    (1) MMTKSLFGVAAFTYFAISSVFAEEAAKPKFTG-------------------LVLDVNKEIIDHVAVESTGIDD-QIATVFAAHRDFAIEKVVDGEKIIKTFDLSKQTPER
BEWA_028210 EMA4    (1) MAARPLFALLSLASWAVCSAVAEEAAKPRPEG-------------------IILDISKEAMDHVTVESTGDEYGVTKTTFTIHEGYAAETVADGSKMISMFNLEFKSPRR
BEWA_042700 EMA5    (1) MIAKSVLGAIALIYLATSGVVSAEKKKKKSEKKGSESKAGK----------ASVNVNKDVIEHVTVTPNEDGT---VVLFTAHEGYSIEKVFEGSTFITKFNLEAYSPKS
BEWA_052710 EMA6    (1) MLSKSFVAILSLACLTVTEASAKNTPKVVG---------------------TVVDISKDAVDHVKIEPEDDGN---TVIFSLKDGYAAEKVVDGSETIKTFDLEKHAPKY
BEWA_016670 EMA7    (1) MV-KSLLRASAVVFLALSGVFAKGDVRYLP---------------------VTVDISNYMLNQPNLFVKVDSE---EVVFNAQEGHAIERVVNGDEVIRTFDLSQEAPKT
BEWA_045350 EMA8    (1) MRAT---SLILVISLAISGTLAAKVHNCSS---------------------ATVNIANDVNEQYNVITKVDEN---HIVYTTAEGYAFKKVVDNLVEVATFDLKATSPKT
BEWA_034050 EMA9    (1) --------------------------------------------------------------------------------------------------------------

                        111                                                                                                          220
BEWA_047350 EMA10 (111) HTCNPKDKLYAALLSPQYSGHAEQLGTGAASVDPVASNKAHVEEAEDKISSSRQEGADPQPGPKPGEGKEEEVGDVGDRDVEIQQKLEEDKPLSLVQASGLPGPPRPETD
BEWA_026850 EMA1   (85) VVRHIKDNKPYVVIAVESALHLVLKKDGD--------------------------------------------------------KWVELEVAEFYQEVLFKGF------
BEWA_017320 EMA2   (86) VTTHLKEDKHYLTVCVVPALHLAFKKEGD--------------------------------------------------------AWVEMPLADLYEQVLFKGR------
BEWA_034340 EMA3   (91) VEKHVKGDKIFVVITVDPALRLAFKKEGD--------------------------------------------------------AWVEMPLADFYEELVFKGL------
BEWA_028210 EMA4   (92) VEKYVVGDRMFVVITVEPALCLSFEKDSG--------------------------------------------------------WWVGIPIYYFYEQVLFKGL------
BEWA_042700 EMA5   (98) VTKHMKNGKIYVTAAVENALHLAYKKDGD--------------------------------------------------------KYEEMPIIDFYEEVLLKGR------
BEWA_052710 EMA6   (87) ITKHIQDGKAYLVIVVEAAYHLAFKKE-K--------------------------------------------------------EWTSIDITAFHESVFFKGF------
BEWA_016670 EMA7   (86) VVKHIDDNYVIVSINVEPSLNLAYKHNGD--------------------------------------------------------RFVEMDITEFYETVFFKGL------
BEWA_045350 EMA8   (84) VTKYLIDDGIYVTVAMEPSQHFAYKKRGN--------------------------------------------------------EYVKLNLVEFYENVLFKDF------
BEWA_034050 EMA9    (1) ----------------------------------------------------------------------------------------------------MKGL------

                        221                                                                                                          330
BEWA_047350 EMA10 (221) QGEDGQADETQGKDTKGAVGEENSTASQHGSGAQNQGHTPTEEPPAATSSTAVDQSQLQPEALGAVELAGQTASTALPEAPQQPDGPSPPANITQGNAQGE//(118)//
BEWA_026850 EMA1  (133) --------EAVSVDLAAAVS-DKFTETTFGSGKKHTFKAPGKRVLKVVDGKTELIDGDNEVVLDLELFVSSDNKVARVVYLYKGDG-----RIKEIFLK-----------
BEWA_017320 EMA2  (134) --------EEVVGDLDKHEDTALFGSEAFGSGKKHTFTATGKKISKVTFGDHVLVDGSYEVFLGLTVYAHGDKKVATVWYLYKPDA-----RIKEVFFE-----------
BEWA_034340 EMA3  (139) --------SGVAVDLDKFADGSLFSAAEFGSGNKHTFSAAGKRASKVTFGEKDLLDGNNEVILDVFVFVSGDKKVAKVVYLYKGDG-----RIKEIFFQ-----------
BEWA_028210 EMA4  (140) --------EGIVIDLEKFADGSLFSAAEFGSGKKHVFKAPGKRILKVVYDENVPLIASEGVVLGLTVYANGEKKIAQVIYIHKEWETRRESRIKEVFFQ-----------
BEWA_042700 EMA5  (146) --------EVATVDLEKLEG-PLFTVTTFGSGKKHTFSSEKKRVGKLVADDDTLIKGEEEFVTELCVFVGGEKSVARVVYLYKGDS-----RIKEVFFQ-----------
BEWA_052710 EMA6  (134) --------ESIVLDLEKFVDSSLFSVEAFGSGEKYAFEAPNKRASKVVSADKDVVSGDDKLILDACVYAKGDSMIATVWYIYKPDG-----RIKEVFFQ-----------
BEWA_016670 EMA7  (134) --------ENVVVDLDAFSTSGYFSSSDFGPGKMYEFEIPSRRISKLVAGNTVLVSGVDELLLGVVVHVNGDRKVATVWYVFKPDG-----RIKEIFFQ-----------
BEWA_045350 EMA8  (132) --------EKVTIDADKCESAVYFDASSFGSGKMYEFPSSLKRPLKVMSAGKQILGVPKEVLIDVVIYVSGSDKILRIGYVSRDDS-----RIKEVFYQ-----------
BEWA_034050 EMA9    (5) --------EAVTIDLD--EPYEEFISSVFGYGKSYSWPFTKKRVGKIVFGEAVILSGKYEVIIELTIFTRGEIKVAKIVYIYKPDD-----RIKEIFYE-----------

                        441                                                        502
BEWA_047350 EMA10 (441) KTDGSAATVVQPAPRVDVTTAAKIRNGMNSSFSADYKAVYDGFSASHVFSAIAVVFAVALFY
BEWA_026850 EMA1  (218) -------LVEKAWKRVEVKDAAETLHGINSTFPADYKVVYDGFSVYGALLAVAAIAFSTLF-
BEWA_017320 EMA2  (220) -------KAGDSWVRVDVTAAAKILNGINPSFSADYKTQYDGFSVYGVFSAVAVVFAVALFY
BEWA_034340 EMA3  (225) -------LVDKAWTRVEVKAAATVLHSIDSSFPADYKTVFDGFSAYGVFSAVAAVFAVALFC
BEWA_028210 EMA4  (231) -------KTEEDWVKVDAKAAATVLHDMNPEFPADYKTVYDGFSVSGVFLAITTILAVALFC
BEWA_042700 EMA5  (231) -------KTEEGWTRVEVDTAAEILHSIDSTFSADYKTIYDGFSAYSVFFAVLAIAFSTVF-
BEWA_052710 EMA6  (220) -------KTEDGWTRVDVTTAAKVLNGMNPDFSTDYKTVYDGFSVSHVFFVAFAIAFSTLFF
BEWA_016670 EMA7  (220) -------LINRTWTRVDTKTAAEALHFINPNFSVGYKPIYDGFSVACVSFTLIAIAFAVLY-
BEWA_045350 EMA8  (218) -------KVRTYWSPVTVSAAAKVLHAMNSAFPADYESLYDGCPVLTVLFSVAALALFVLFQ
BEWA_034050 EMA9   (89) -------RRSDDWVRVDIPIAAKVINKIEPFFSVDYKTIYDGFSAYGVFSVVIATFALVLFH
```
