# Supplementary material for: Comparative genomic analysis and phylogenetic position of Theileria equi
Source: BMC Genomics. 2012 Nov 9;13:603. doi: 10.1186/1471-2164-13-603 (PMC3505731; doi:10.1186/1471-2164-13-603)
Supplement: Additional file 7 — Table. Proteins used in phylogenetic analysis. [file 1471-2164-13-603-S7.pdf]

Table: Proteins used in phylogenetic analysis

Gene model	Putative function	*T. equi* length (aa)	Alignment length (aa)	PI[A]	# MPT[B]	Topology[C]
BEWA_028760[D]	*myosin A*	*835*	*847*	*409*	*1*	*A*
BEWA_005330	*eukaryotic translation initiation factor*	*733*	*789*	*401*	*1*	*A*
BEWA_037430	*DNA replication licensing factor MCM5*	*779*	*859*	*322*	*1*	*A*
BEWA_049210	*adenosylhomocysteinase*	*492*	*507*	*306*	*1*	*A*
BEWA_009450	*heat shock protein 70kD*	*654*	*671*	*301*	*1*	*A*
BEWA_017580	*vacuolar ATP synthase subunit C*	*406*	*453*	*263*	*1*	*A*
BEWA_043900	*protein kinase domain containing protein*	*503*	*542*	*239*	*1*	*A*
BEWA_037580	*DnaJ domain containing protein*	*420*	*455*	*230*	*1*	*A*
BEWA_019520	*heat shock protein 90 (HSP90)*	*717*	*766*	*220*	*1*	*A*
BEWA_032130	*chaperonin-60kD, ch60*	*534*	*559*	*208*	*1*	*A*
BEWA_041190	*cell division cycle protein 48*	*804*	*858*	*193*	*1*	*A*
BEWA_037520	*clathrin-adaptor chain*	*441*	*492*	*192*	*1*	*A*
BEWA_034850	*conserved hypotheticalprotein*	*202*	*254*	*152*	*1*	*A*
BEWA_015100	*proteasome subunit alpha type*	*260*	*274*	*150*	*1*	*A*
BEWA_022370	*vacuolar ATP synthase subunit D*	*237*	*259*	*139*	*1*	*A*
BEWA_009590	*enolase*	*443*	*479*	*135*	*1*	*A*
BEWA_031590	*60S acidic ribosomal protein P0*	*314*	*326*	*134*	*1*	*A*
BEWA_017550	*histone deacetylase*	*449*	*472*	*132*	*1*	*A*
BEWA_045860	*adenylate kinase*	*246*	*262*	*125*	*1*	*A*
BEWA_010440	*vacuolar sorting protein 26*	*272*	*330*	*124*	*1*	*A*
BEWA_038400	*eukaryotic translation initiation factor 4A*	*392*	*396*	*123*	*1*	*A*
BEWA_045060	*protein transport protein Sec61*	*477*	*481*	*122*	*1*	*A*
BEWA_011020	*40S ribosomal protein S7*	*193*	*217*	*113*	*1*	*A*
BEWA_027970	*conserved hypothetical protein*	*194*	*222*	*113*	*1*	*A*
BEWA_019150	*proteasome regulatory subunit*	*312*	*315*	*112*	*1*	*A*
BEWA_010900	*vacuolar proton-translocating ATPase*	*181*	*217*	*109*	*1*	*A*
BEWA_010860	*proteasome subunit alpha type*	*238*	*258*	*106*	*1*	*A*
BEWA_052280	*Ras-related protein Rab1b (small GTP-binding protein)*	*221*	*230*	*98*	*1*	*A*
BEWA_052650	*40S ribosomal protein S19*	*173*	*173*	*96*	*1*	*A*
BEWA_052880	*40S ribosomal protein S3a*	*263*	*265*	*94*	*1*	*A*
BEWA_042640	*60S ribosomal protein L2/L8*	*260*	*261*	*93*	*1*	*A*
BEWA_031840	*translationally controlled tumor protein*	*175*	*175*	*90*	*1*	*A*
BEWA_027590	*60S ribosomal protein L18a*	*190*	*193*	*89*	*1*	*A*
BEWA_053410	*60S ribosomal protein L28*	*138*	*168*	*88*	*1*	*A*
BEWA_030290	*60S ribosomal protein L6*	*190*	*190*	*87*	*1*	*A*
BEWA_021630	*nucleoside diphosphate kinase*	*150*	*175*	*86*	*1*	*A*
BEWA_038020	*protein kinase domain containing protein*	*290*	*302*	*81*	*1*	*A*
BEWA_018030	*60S ribosomal protein L27e*	*149*	*165*	*76*	*1*	*A*
BEWA_024780	*eukaryotic translation initiation factor 2, beta subunit*	*243*	*248*	*76*	*1*	*A*
BEWA_032210	*DNA-directed RNA polymerase subunit rpb8*	*146*	*151*	*73*	*1*	*A*

BEWA_009670	*60S acidic ribosomal protein P1*	*116*	*123*	*72*	*1*	*A*
BEWA_044990	*multiprotein bridging factor type*	*147*	*160*	*71*	*1*	*A*
BEWA_020290	*60S ribosomal protein L27a*	*145*	*158*	*56*	*1*	*A*
BEWA_006560	*60S ribosomal protein L22*	*123*	*45*	*55*	*1*	*A*
BEWA_008940	*40S ribosomal proteinS3*	*219*	*236*	*55*	*1*	*A*
BEWA_030910	*60S ribosomal protein L11*	*172*	*175*	*48*	*1*	*A*
BEWA_026740	*translation initiation factor eIF-1A*	*154*	*176*	*44*	*1*	*A*
BEWA_014410	*60S ribosomal protein L37a*	*95*	*96*	*36*	*1*	*A*
BEWA_005410	*40S ribosomal protein S14*	*152*	*173*	*31*	*1*	*A*
BEWA_028110	*histone H2b*	*124*	*131*	*22*	*1*	*A*
BEWA_025390	conserved hypothetical protein	165	198	115	1	B
BEWA_038070	ubiquitin-conjugating enzyme family member protein	198	219	76	1	B
BEWA_026400	eukaryotic peptide chain release factor	428	446	137	1	B
BEWA_009700	vacuolar ATP synthase catalytic subunit A	612	627	241	1	B
BEWA_027570	60S ribosomal protein L18	184	229	65	1	B
BEWA_009410	conserved hypothetical protein	248	278	180	1	B
BEWA_013610	40S ribosomal protein S4	184	206	79	1	B
BEWA_051440	40S ribosomal protein S8	191	223	76	1	B
BEWA_007100	pyruvate kinase	516	544	249	1	B
BEWA_045850	diphthinesynthase	271	320	131	1	B
BEWA_032470	prohibitin	279	318	85	1	B
BEWA_011610	importin alpha	532	583	301	1	B
BEWA_005460	60S ribosomal protein L12	166	169	57	1	B
BEWA_031600	60S ribosomal protein L38	79	101	53	1	B
BEWA_006190	LSM domain containing protein	122	161	64	1	B
BEWA_012260	60S ribosomal protein L34a	145	162	83	1	B
BEWA_020360	60S ribosomal protein L23	140	146	26	1	B
BEWA_009500	phosphoglycerate mutase	250	303	117	1	B
BEWA_016650	40S ribosomal protein S27	83	86	24	1	B
BEWA_007700	ADP-ribosylation factor family member protein	196	219	82	1	B
BEWA_027890	conserved hypothetical protein	199	211	144	1	B
BEWA_010040	40S ribosomal protein	280	296	113	1	B
BEWA_020160	60S ribosomalprotein L30	109	111	43	1	B
BEWA_038930	actin	382	430	209	1	B
BEWA_011640	DNA-directed RNA polymerase	206	214	66	1	B
BEWA_031870	small GTP-binding protein domain containing protein	367	368	85	1	B
BEWA_019950	chaperonin containing t-complex protein 1, epsilon subunit	536	575	234	1	B
BEWA_040690	conserved hypothetical protein	76	113	52	1	B
BEWA_005070	60S ribosomal protein L36	117	120	63	1	B
BEWA_049420	60S ribosomal protein L19	180	201	74	1	C
BEWA_024880	1-acyl-sn-glycerol-3-phosphate acyltransferase	276	314	173	1	C
BEWA_018100	60S ribosomal protein L5e	307	326	152	1	C
BEWA_010270	60S ribosomal protein L26	138	175	51	1	C

BEWA_019170	conserved hypothetical protein	173	201	107	1	C
BEWA_008800	vacuolar ATP synthase subunit	385	430	206	1	C
BEWA_010850	proteasome subunit alpha type	256	272	104	1	C
BEWA_025460	60S ribosomal protein L17	188	209	87	1	C
BEWA_016270	60S ribosomal protein L24	178	212	100	1	C
BEWA_014640	glucose-6-phosphate isomerase	555	615	301	1	C
BEWA_005310	40S ribosomal protein S24	136	160	61	1	C
BEWA_011460	conserved hypothetical protein	130	145	58	1	C
BEWA_030920	40S ribosomal protein S18	155	156	49	1	C
BEWA_024030	proliferating cell nuclear antigen 2	268	305	134	1	D
BEWA_042550	ubiquitin domain containing protein	91	125	30	1	D
BEWA_001300	26S protease regulatory subunit	397	412	112	1	D
BEWA_003280	60S ribosomal protein L1	218	221	82	1	D
BEWA_043560	chaperonin containing t-complex protein1, theta subunit	539	591	329	1	D
BEWA_008860	conserved hypothetical protein	180	199	116	1	D
BEWA_026680	DNA-directed RNA polymerase I	316	385	172	1	D
BEWA_039360	U6 small nuclear ribonucleoprotein E	89	134	46	1	E
BEWA_050030	chaperonin containing t-complex protein 1, alpha subunit	547	574	226	1	E
BEWA_054840	60S ribosomal protein L35a	118	146	58	1	F
BEWA_018170	transcription factor btf3	162	227	95	1	F
BEWA_006940	N-acetyltransferase	153	192	104	1	G
BEWA_019530	ADP-ribosylation factor put.	182	189	65	1	G
BEWA_051470	chaperonin-60kD, ch60	540	573	279	1	G
BEWA_020790	signal peptide containing protein	412	466	258	1	G
BEWA_010610	conserved hypothetical protein	279	313	120	1	H
BEWA_027050	40S ribosomal protein S2	262	292	71	1	H
BEWA_007000	proteasome subunit beta type 3	218	244	101	1	I
BEWA_013620	40S ribosomal protein S28	68	68	13	1	J
BEWA_011470	S-adenosylmethionine synthetase	401	435	167	2	AB
BEWA_006110	proteasome subunit alpha type	277	301	136	2	AB
BEWA_052640	40S ribosomal protein S12	143	170	67	2	AB
BEWA_040030	eukaryotic translation initiation factor 2 alpha subunit	331	366	136	2	AB
BEWA_030470	conserved hypothetical protein	258	367	163	2	AB
BEWA_031760	conserved hypothetical protein	118	154	76	2	AB
BEWA_019430	60S ribosomal protein L37	92	103	32	2	AB
BEWA_037530	26S proteasome regulatory subunit 7	426	460	107	2	AC
BEWA_018280	U6 small nuclear ribonucleoprotein	94	98	50	2	AD
BEWA_039830	26S proteasome subunit 4	440	457	90	2	AC
BEWA_017900	40S ribosomal protein S8	131	137	31	2	AD
BEWA_020460	translation initiation factor 6	250	280	90	2	AC
BEWA_031130	bet3 transport protein	186	206	88	2	AD
BEWA_024530	splicing factor 3A subunit 2	238	256	124	2	AC
BEWA_010820	er lumen protein retaining receptor	224	223	88	2	AD

BEWA_013500	replication factor C subunit 4	325	357	147	2	AC
BEWA_042350	60S ribosomal protein L10e	229	235	152	2	AC
BEWA_017790	nuclear transport factor 2	125	169	74	2	BG
BEWA_000870	40S ribosomal protein S15	149	152	53	2	BC
BEWA_052490	60S ribosomal protein L7	259	268	111	2	BC
BEWA_010950	40S ribosomal protein S10	113	155	58	2	BO
BEWA_020010	conserved hypothetical protein	188	246	94	2	BC
BEWA_034410	DnaJ domain containing protein	388	409	244	2	BF
BEWA_041030	actin depolymerizing factor	120	136	92	2	EH
BEWA_052070	60S ribosomal protein L15	206	222	56	2	DE
BEWA_052600	small nuclear ribonucleoprotein G	105	111	50	2	CN
BEWA_014000	prohibitin	273	296	95	2	CD
BEWA_002990	60S ribosomal protein L32	133	137	69	2	DE
BEWA_041920	ribosomal protein S26	115	115	36	2	DF
BEWA_013830	U6 snRNA-associated Sm-like protein LSm8	95	115	53	2	CR
BEWA_044960	40S ribosomal protein S5	194	207	37	3	ABD
BEWA_018110	60S ribosomal protein L7a	270	289	134	3	ACD
BEWA_021800	vacuolar ATP synthase subunit f	123	141	56	3	ACE
BEWA_028610	40S ribosomal protein S13e	152	154	46	3	CPQ
BEWA_018190	60S ribosomal protein L10	229	228	62	3	CEI
BEWA_041010	60S ribosomal protein L31	119	120	59	3	CDF
BEWA_024170	histone H2a	153	164	24	3	DFG
BEWA_010100	ribosomal protein S27	165	168	90	3	DFG
BEWA_041310	small ribonucleoprotein D1	120	148	46	7	ADEHKLM

[A] Number of parsimony informative characters (calculated using PAUP*).
[B] Number of equally parsimonious trees recovered.
[C] See Figure S3 for tree topologies A-D.
[D] Protein sequences producing most commonly recovered topology, sorted by parsimony informative characters are boxed.
